# Caribbean fish feces are an environmental hotspot of viable Symbiodiniaceae

**DOI:** 10.3389/fmicb.2025.1715855

**Published:** 2026-02-12

**Authors:** K. R. Titus, R. Castellon, C. Washington, J. Cooper, C. Grupstra, J. Bloomberg, S. R. Coy, B. H. Farmer, C. E. Karrick, S. Meiling, J. Quetel, A. M. Rossin, A. Veglia, J. Watkins, K. Evans, A. Apprill, D. M. Holstein, L. Mydlarz, M. Brandt, A. M. S. Correa

**Affiliations:** 1 Ecology and Evolutionary Biology, Rice University, Houston, TX, United States; 2 Biological Sciences, Florida Atlantic University, Boca Raton, FL, United States; 3 Woods Hole Oceanographic Institute, Falmouth, MA, United States; 4 Massachusetts Institute of Technology, Cambridge, MA, United States; 5 Louisiana State University, Baton Rouge, LA, United States; 6 University of California, Berkeley, CA, United States; 7 University of the Virgin Islands, St. Thomas, VI, United States; 8 Cooperative Institute of Marine and Atmospheric Science, Miami, FL, United States; 9 University of Puerto Rico-Mayaguez, Mayaguez, PR, United States; 10 Stony Brook University, Stony Brook, NY, United States; 11 University of Texas, Arlington, TX, United States

**Keywords:** reef fish, Symbiodiniaceae communities, Symbiodiniaceae density, symbiosis, trophic transmission

## Abstract

Approximately 85% of stony coral species initially acquire their nutritional symbionts (Family Symbiodiniaceae) from the environment (horizontal transmission). Recent studies have identified live Symbiodiniaceae cells in the feces of coral-eating (corallivorous) and herbivore/detritivore fish, and thus these fish could vector Symbiodiniaceae to prospective stony coral hosts. However, nearly all data on viable Symbiodiniaceae cell densities in fish feces are from Pacific reefs. This study quantifies the density and diversity of viable Symbiodiniaceae cells in the feces of six Caribbean corallivore and herbivore/detritivore fish species in the U.S. Virgin Islands, enabling comparisons of consumer-symbiont pathways between ocean basins. Caribbean fish feces contained an average of 5 million viable Symbiodiniaceae cells ml^−1^, comparable to previously reported values for Pacific corallivores. However, unlike on Pacific reefs, where Symbiodiniaceae cell densities varied in feces by fish trophic group, in the Caribbean, high densities of Symbiodiniaceae cells were documented in fish feces across feeding categories. In Caribbean herbivore/detritivore feces, high Symbiodiniaceae densities likely reflect observed, yet unexpected, feeding by these fishes on corals. Contributions of sloughed diseased coral tissue to detritus on U.S. Virgin Islands reefs may have also increased the number of Symbiodiniaceae cells consumed by detritivorous fishes. Symbiodiniaceae genera *Symbiodinium*, *Breviolum*, *Cladocopium*, *Durusdinium*, and *Fugacium* were detected in Caribbean fish feces. These findings demonstrate that corallivore and herbivore/detritivore fish feces constitute environmental hotspots of viable Symbiodiniaceae on Caribbean reefs.

## Introduction

The majority (~85%) of shallow-water, reef-building corals establish endosymbiotic partnerships with dinoflagellates in the family Symbiodiniaceae through horizontal transmission (i.e., acquisition of symbionts from the surrounding environment). Although the Symbiodiniaceae diversity harbored by a given coral species is non-random and selective, once established, symbiont communities within individual corals can shift based on environmental conditions and symbiont availability (Davies et al., 2023; Bright and Bulgheresi, 2010; Harrison and Wallace, 1990; Kenkel and Bay, 2018). Various sources, including the water column (Scharfenstein et al., 2022; Williamson et al., 2021), sediments (Ali et al., 2019), macroalgae (Littman et al., 2008) and consumer feces (Castro-Sanguino and Sánchez, 2012; Grupstra et al., 2021; Parker, 1984) have been identified as environmental reservoirs of Symbiodiniaceae. Understanding the properties and dynamics of these symbiont reservoirs is essential for predicting coral resilience in a changing ocean.

Recent work on nine Pacific reef fish species, spanning obligate and facultative corallivores, as well as herbivores/detritivores, demonstrated that fish feces are a significant source of viable, photosynthetically active Symbiodiniaceae (Grupstra et al., 2021). *Cladocopium* and *Durusdinium*, the two Symbiodiniaceae genera most frequently harbored by Pacific coral species, dominated the feces of these fish. Obligate corallivore feces harbored the highest densities of Symbiodiniaceae, containing 5–7 orders of magnitude more cells than found in the water column or adjacent sediments. Corallivorous fishes were estimated to disperse over 100 million viable symbiont cells per 100 m^2^ per day, highlighting the importance of consumers in distributing symbionts around coral reefs (Grupstra et al., 2021).

Despite growing evidence that reef fish trophically transmit key coral symbionts on Pacific reefs, little is known about the density and diversity of Symbiodiniaceae in the feces of Caribbean reef fishes (but see Castro-Sanguino and Sánchez, 2012). This knowledge gap could lead to problematic overestimates of Symbiodiniaceae density and underestimates of diversity in Caribbean fish feces, given regional differences in coral and fish community structure. For example, obligate and facultative corallivore fish species are found on Pacific reefs, whereas Caribbean reefs harbor only facultative corallivores (Pratchett et al., 2013). Since obligate corallivores eat mostly coral, their feces are expected to contain higher Symbiodiniaceae densities. Furthermore, South Pacific reefs generally support higher coral cover than Caribbean reefs, potentially augmenting facultative corallivore feces with the incidental consumption of corals on Pacific reefs, leading to higher densities in fish feces (McManus et al., 2021). Published characterizations of Symbiodiniaceae in Caribbean reef fish feces are limited to a single facultative corallivore species, the parrotfish *Sparisoma viride*. The densities of Symbiodiniaceae in *S. viride* feces reported by Castro-Sanguino and Sánchez (2012) were 1–3 orders of magnitude lower than those documented in Pacific corallivore feces (Grupstra et al., 2021); no data are available for Caribbean herbivore/detritivores.

Pacific stony corals are typically dominated by Symbiodiniaceae lineages in the genera *Cladocopium* and *Durusdinium*, though *Symbiodinium* may also occur in high abundance (Cunning et al., 2024; LaJeunesse et al., 2003). In contrast, Caribbean stony corals frequently harbor Symbiodiniaceae representing four genera *Symbiodinium*, *Breviolum*, *Cladocopium*, and *Durusdinium* (LaJeunesse et al., 2003; Silverstein et al., 2012). *Fugacium* and *Gerakladium*, two other genera of Symbiodiniaceae, are less frequently detected from stony corals. Some lineages within each genus may have a free-living lifestyle; other *Fugacium* lineages are most commonly found in foraminifera, whereas some lineages of *Gerakladium* are harbored by excavating sponges (Clionaidae) (LaJeunesse et al., 2018; Mote et al., 2021; Million et al., 2025). Castro-Sanguino and Sánchez (2012) reported *Symbiodinium*, *Breviolum*, and *Gerakladium* from *S. viride* feces, whereas Grupstra et al. (2021) reported predominantly *Cladocopium* and *Durusdinium* (but also *Symbiodinium* and *Fugacium*) from Pacific fish feces. The total diversity of Symbiodiniaceae detected from the feces of Caribbean reef fish is likely to increase with broader taxonomic sampling. Caribbean reef fish communities exhibit lower species diversity across trophic groups (Harmelin-Vivien, 2002); more generalist species contribute to higher functional redundancy (Floeter et al., 2004; Mouillot et al., 2014). This could contribute to greater similarity in Symbiodiniaceae assemblages across the feces of Caribbean reef fish, relative to assemblages in Pacific fish feces.

Understanding the role of regional fish communities in the trophic transmission of Symbiodiniaceae is essential for advancing ecological theory and conservation strategies. Here, we quantify the density and diversity of Symbiodiniaceae in the feces of six Caribbean reef fish species and compare aspects of consumer-symbiont pathways across ocean basins.

## Methods

### Benthic composition surveys

In October 2021 and April 2022, benthic composition surveys were conducted on two reefs (10–15 m depth) in St. John, U.S. Virgin Islands (USVI, Coral Bay, South Haulover Bay) and two reefs (10–25 m depth) in St. Croix, USVI (Buck Island, Cane Bay; Supplementary Figure 1). Three 10-m video transects were recorded at each site; videos were clipped into non-overlapping images (average *n* = 16 images per transect). Thirty random points were assigned to each image and the benthic organism under each point was identified. Percent cover was calculated for each organismal category based on the total points identified per transect (on average, *n* = 436 points could be identified per transect). Raw (unclipped, no random points applied) video transect footage was used to calculate the mean disease prevalence of observed corals across each site.

### Fish follows and fecal sampling

In April 2022, divers conducted 5-min focal observations (or follows) across the four reef sites to estimate the foraging behaviors of six prominent Caribbean facultative corallivores and herbivore/detritivore fishes. Facultative corallivores typically eat hard and soft corals, as well as sponges, algaes, and benthic invertebrates while herbivores/detritivores have been observed to forage on algaes and detritus. Follows were conducted on a minimum of eight individuals per island for each of the six Caribbean reef fishes, except for *Chaetodon striatus*, which was only observed at St. Croix reef sites. Fish follows were focused on two species of facultative corallivorous butterflyfish (*Chaetodon capistratus* and *Chaetodon striatus*), two species of facultative corallivorous parrotfish (*Sparisoma aurofrenatum* and *Sparisoma viride*), and two species of herbivorous/detritivorous surgeonfish (*Acanthurus bahianus* and *Acanthurus coeruleus*). Upon fish species identification, phase (initial or terminal), length, social status (single, pair, group), and foraging behaviors (number of bites, substrate type, substrate species, substrate health state) were recorded. After all follows were completed, a minimum of six adult or terminal phase individuals for each of the six fish species were speared, measured, photographed, and dissected for fecal content (retrieved from the hindgut).

### Symbiodiniaceae cell density and viability

To quantify Symbiodiniaceae cells in fish feces, we used protocols similar to those established by Grupstra et al. (2021). Each fecal sample (*n* = 56) was weighed and fixed in 750 uL of 10% seawater formalin, homogenized, filtered, and stained with trypan blue (5:1 sample to stain; Corning, Manassas, VA). After staining, live and dead Symbiodiniaceae cell densities were counted using light microscopy (20X) with a Neubauer hemocytometer in a dark room. Individual cells were considered to have been alive at the time of fixation if they maintained a golden-brown color despite exposure to trypan blue stain; cells that stained blue were interpreted as being dead at the time of fixation, based on having a disrupted cell wall. Using the Symbiodiniaceae counts and the weight of the fish feces, and assuming that the densities of the sampled feces were equal to that of water (mass/volume: 1 g/mL), the number of living and dead Symbiodiniaceae cells per ml feces were calculated per sample. Density of Symbiodiniaceae cells per ml feces could not be calculated for some samples (4 *C. capistratus*, 2 *C. striatus*, 2 *S. aurofrenatum*, 5 *A. bahianus*) due to lack of access to a scale during fecal collections at some remote sites, leaving 43 fecal samples for Symbiodiniaceae density analyses (Supplementary Figure 1B).

### Symbiodiniaceae diversity

Symbiodiniaceae diversity in fish feces (*n* = 56) was compared to that in local coral tissues (*n* = 244) following collection, preservation, DNA extraction, and sequencing. Coral species initially flash frozen and later airbrushed with 10X PBS included *Acropora cervicornis* (*n* = 5), *Agaricia agaricites* (*n* = 44), *Colpophyllia natans* (*n* = 52), *Diploria labyrinthiformis* (*n* = 25), *Montastraea cavernosa* (*n* = 39), *Orbicella annularis* (*n* = 36), and *Porites astreoides* (*n* = 43). Aliquots of each fecal sample were preserved in 750 uL of DNA/RNA shield (Zymo Research, CA). DNA was extracted from corals and fish feces using Qiagen DNeasy PowerBiofilm Kits (Qiagen, Aarhus, Denmark). The internal transcribed spacer-2 (ITS-2) region of Symbiodiniaceae rDNA was sequenced from all DNA following protocols in Howe-Kerr et al., (2020) using the primers SYM_VAR_5.8S2 (5’-GAATTGCAGAACTCCGTGAACC-3′) and SYM_VAR_REV (5’-CGGGTTCWCTTGTYTGACTTCATGC-3′; Hume et al., 2018). DNA was sequenced on the Illumina MiSeq platform at the Center for Quantitative Life Sciences (Oregon State University). The resulting sequence data were processed using the online SymPortal platform (Hume et al., 2019) and samples with low reads (fecal samples with <400 reads = 10) were discarded, leaving 46 fish fecal samples for downstream analyses (Supplementary Figure 1B). Symportal identified 121 ITS-2 type profiles from sequenced reads, many of which shared a dominant DIV (defining intragenomic variant) or were highly similar to another type profile (e.g., A3-A3t-A3ad vs. A3-A3t-A3ad-A3ac). The multi-copy nature of the ITS-2 can straddle inter- and intra-specific variation, posing challenges for interpretation (Davies et al., 2023). Therefore, to be conservative in some comparisons, we identified corals and fish feces that contained the same dominant DIVs (Hume et al., 2018; Supplementary Table 1).

### Statistical analyses

A permutational multivariate analysis of variance (PERMANOVA) and Dunn’s *post hoc* test with Bonferroni-corrected *p*-values were used to assess the benthic compositions of the four reefs and the two islands. To test for differences in foraging behaviors (total bites recorded) across the six fish species, a Kruskal–Wallis and Dunn’s *post hoc* test with Benjamin Hochberg-corrected *p*-values for multiple comparisons was performed using dunn.test package (Dinno and Dinno, 2017), as well as a multivariate analysis of variance (MANOVA), in R version 4.2.2 (R Core Team, 2022). To test for differences in Symbiodiniaceae densities among the feces of fish species and foraging groups, outliers for live and dead Symbiodiniaceae cells were identified and removed using rstatix package version 0.7.2 (Kassambara, 2023). A Wilcox test followed by Kruskal–Wallis and Dunn’s *post hoc* tests with Benjamin Hochberg-corrected *p*-values were conducted to assess for effects of foraging group (facultative corallivore, herbivore) or fish Family (butterflyfish, parrotfish, surgeonfish) on live and dead Symbiodiniaceae cell densities. A Wilcoxon signed-rank test was used to identify differences in live and dead Symbiodiniaceae cell densities in the feces of different fish species.

The approaches used here to quantify Symbiodiniaceae density and diversity have also been applied to Pacific reef organisms, including butterflyfishes (*Chaetodon citrinellus*, *Chaetodon lunulatus*, *Chaetodon ornatissimus*, *Chaetodon pelewensis*, and *Chaetodon reticulatus*), a filefish (*Amanses scopus*), a parrotfish (*Chlorurus spilurus*), and surgeonfishes (*Ctenochaetus flavicauda* and *Ctenochaetus striatus*). These Pacific fish species span obligate corallivore (*A. scopus*, *C. lunulatus*, *C. ornatissimus* and *C. reticulatus*), facultative corallivore (*C. citrinellus*, *C. pelewensis*, and *C. spilurus*) and herbivore/detritivore (*C. flavicauda* and *C. striatus*) foraging groups. To compare the Symbiodiniaceae cell densities found in the feces of Caribbean fishes to those in previously studied Pacific fishes (Grupstra et al., 2021), a pairwise dunn-test with Benjamin Hochberg-corrected *p*-values was used with foraging group and geographic region as the two categorical independent variables. Assumptions of normality and homogeneity of variance were tested using Shapiro tests and the Levene’s test. To provide context for Symbiodiniaceae cell density comparisons between feces of Caribbean and Pacific fishes, we calculated percent coral bites and other benthic substrates from Pacific fish foraging surveys conducted by Grupstra et al. (2021). For details on how Pacific fish foraging surveys were conducted, see Grupstra et al. (2021) (Supplementary Table 3); survey methodologies were consistent with those used in the present study.

To determine the extent to which Symbiodiniaceae assemblages (from the 65 dominant ITS-2 DIVs; Supplementary Table 1) differed across fish species and foraging groups, a PERMANOVA was conducted using the adonis2 package (Heine, 2014). These analyses were followed with a pairwise PERMANOVA using the pairwise.adonis package (Heine, 2014). When beginning the analyses on the assemblages of Symbiodiniaceae found in fish feces versus the communities detected in coral tissues, a significant amount of dispersion was revealed using betadisper() in the vegan package (Anderson, 2006). PERMANOVA is highly sensitive to heterogeneous dispersion with an unbalanced design (Anderson and Walsh, 2013), so six samples were randomly sub-sampled for each coral and fish species (*A. agaricites*: 31, 45, 252, 257, 434, 2097; *A. bahianus:* 2,209, 2,225, 2,226, 2,234, 2,235, 2,237; *A. cervicornis*: 66, 70, 395, 1,151, 1,160 (only 5 samples); *A. coeruleus*: 2,201, 2,202, 2,205, 2,211, 2,240, 2,242; *C. capistratus*: 2,213, 2,223, 2,229, 2,230, 2,231, 2,251; *C. natans*: 119, 241, 317, 379, 383, 2025; *C. striatus*: 2,227, 2,248, 2,249, 2,250, 2,253; *D. labyrinthiformis*: 12, 288, 365, 414, 604, 610; *M. cavernosa*: 81, 357, 527, 542, 653, 1,366, 2011; *O. annularis*: 91, 134, 598, 20,288, 2,065, H126; *P. astreoides*: 190, 216, 341, 4,288, 648, 2080; *S. aurofrenatum*: 2,210, 2,215, 2,216, 2,239, 2,244, 2,255; *S. viride*: 2,206, 2,208, 2,219, 2,221, 2,222, 2,252). Subsampling balanced the sampling design between the fish fecal and coral tissue samples. A PERMANOVA and pairwise PERMANOVA were then run on the sub-sampled data. All figures were created using ggplot2 (Wickham et al., 2016) and viridisLite (Garnier et al., 2023) packages.

## Results

### USVI reefs vary in benthic cover

South Haulover (St. John) had substantial gorgonian, macroalgae, and stony coral cover (averaging 21% (SD ± 4.9), 19% (SD ± 8.1), and 15% (SD ± 4.2), respectively; Supplementary Figure 2A). Stony coral cover (14% ± 4.3) was the dominant living cover at Coral Bay (St. John), but other substrates (e.g., epilithic algae, anthropogenic debris, unknown) were most common (62% ± 7.8). Buck Island (St. Croix) had high rock/sand, gorgonian and macroalgae cover (averaging 30% (SD ± 3.5), 22% (SD ± 4.7), and 17% (SD ± 6.7), respectively). Cane Bay (St. Croix) had high macroalgae and stony coral cover (averaging 35% (SD ± 4.5) and 16% (SD ± 3.9), respectively). The benthic compositions of the four USVI reef sites significantly differed (PERMANOVA: df = 3.20, *F* = 15.992, *p* = 0.001; Supplementary Figure 2B). No diseased corals were observed from the non-overlapping images clipped from the video transect surveys. However, based on the raw video transect footage, mean disease prevalence on observed corals at Buck Island was *M* = 2.58% ± 0.9 (St. Croix; Supplementary Figure 2A), Cane Bay was *M* = 0.71% ± 0.5 (St. Croix), and South Haulover was *M* = 3.97% ± 1.3 (St. John). Quantitative disease results are not available for Coral Bay (St. John), but prevalence was comparable to South Haulover (Brandt, Meiling *pers. obs.*).

### Caribbean fishes primarily bite rock/sand, algae substrates, and stony corals

**Figure 1:**
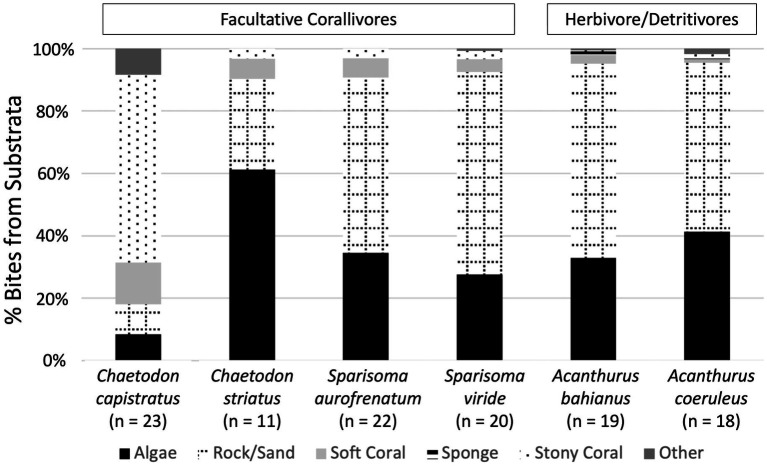
Percent bites observed on the six Caribbean fish species across reef substrate types during fish follows. Five-minute focal surveys were conducted on two species of facultative corallivorous butterflyfish (*Chaetodon capistratus* and *Chaetodon striatus*), two species of facultative corallivorous parrotfish (*Sparisoma aurofrenatum* and *Sparisoma viride*), and two species of herbivorous/detritivorous surgeonfish (*Acanthurus bahianus* and *Acanthurus coeruleus*).

A total of 4,361 bites were taken by 113 individuals during fish follows (Supplementary Table 2). Herbivorous surgeonfishes took the most bites on average per five-minute survey (*A. bahianus* averaged 73 bites and *A. coeruleus* averaged 42 bites), followed by corallivorous parrotfish (*S. viride* averaged 40 bites and *S. aurofrenatum* averaged 38 bites), then corallivorous butterflyfish (*C. capistratus* averaged 23 bites and *C. striatus* averaged 6 bites). Surgeonfish and parrotfish took bites mostly from rock/sand (54.1–65% of bites across species) and algae (27.6–41.4% of bites across species, Figure 1, Supplementary Table 2). Butterflyfish varied: *C. capistratus* mainly bit stony coral substrates (60.2% of bites) and *C. striatus* mainly bit algal substrates (61.3% of bites). The six species of Caribbean fishes took bites from gorgonians (total bites = 208), as well as 12 stony coral species (total bites = 378), primarily *O. annularis* (total bites = 150), *Orbicella franksi* (total bites = 27), *Millepora* sp. (total bites = 20), and *P. astreoides* (total bites = 16, Supplementary Table 2). The number of bites taken across fish species differed (Kruskal–Wallis test: *X*^2^ = 14.267, df = 5, *p* = 0.014; Supplementary Table 4). *Chaetodon striatus* foraged significantly less; individuals of this species averaged 6 bites per follow, whereas for all other species in this study, average bites per follow ranged from 23 to 73. Fish showed no preferences for a particular coral health state (apparently healthy vs. diseased; Kruskal–Wallis test: *X*^2^ = 8.1707, df = 10, *p* = 0.612); however, a majority of bites were on visually healthy corals (303 total bites on apparently healthy corals; 32 total bites on apparently healthy tissue on a diseased coral; 43 bites on diseased coral tissue). Given that disease prevalence was <4% across the four sites, these foraging patterns might indicate a preference for diseased corals, particularly by *C. capistratus*.

### Fish families vary in their defecation rates and fates

A total of 64 fish defecations were recorded across all species (Supplementary Table 2). The average number of defecations per five-minute fish follow differed among the fish families (Kruskal–Wallis test: *X*^2^ = 19.73, df = 2, *p* < 0.001) and individual species (Kruskal–Wallis test: *X*^2^ = 21.6992, df = 5, *p* < 0.001; Supplementary Table 5). Parrotfish accounted for 73.4% (47 of 64) of observed defecations; 18.8% (12 of 64) were by surgeonfish, and 7.8% (5 of 64) were by butterflyfish (Supplementary Table 2). All the butterflyfish defecations observed were by *C. capistratus* and landed on substrates that were not hard coral, rock/sand, or macroalgae. A majority of defecations from parrotfish fell on rock/sand substrates (27 of 47), however, 8 defecations were recorded to fall on hard coral substrates, 7 defecations on algal substrates, and 5 on other substrates. Surgeonfish defecations landed on hard corals (5 of 12), rock/sand (5 of 12) and other substrates (2 of 12).

### High Symbiodiniaceae cell densities in Caribbean corallivorous and herbivorous fish feces

**Figure 2:**
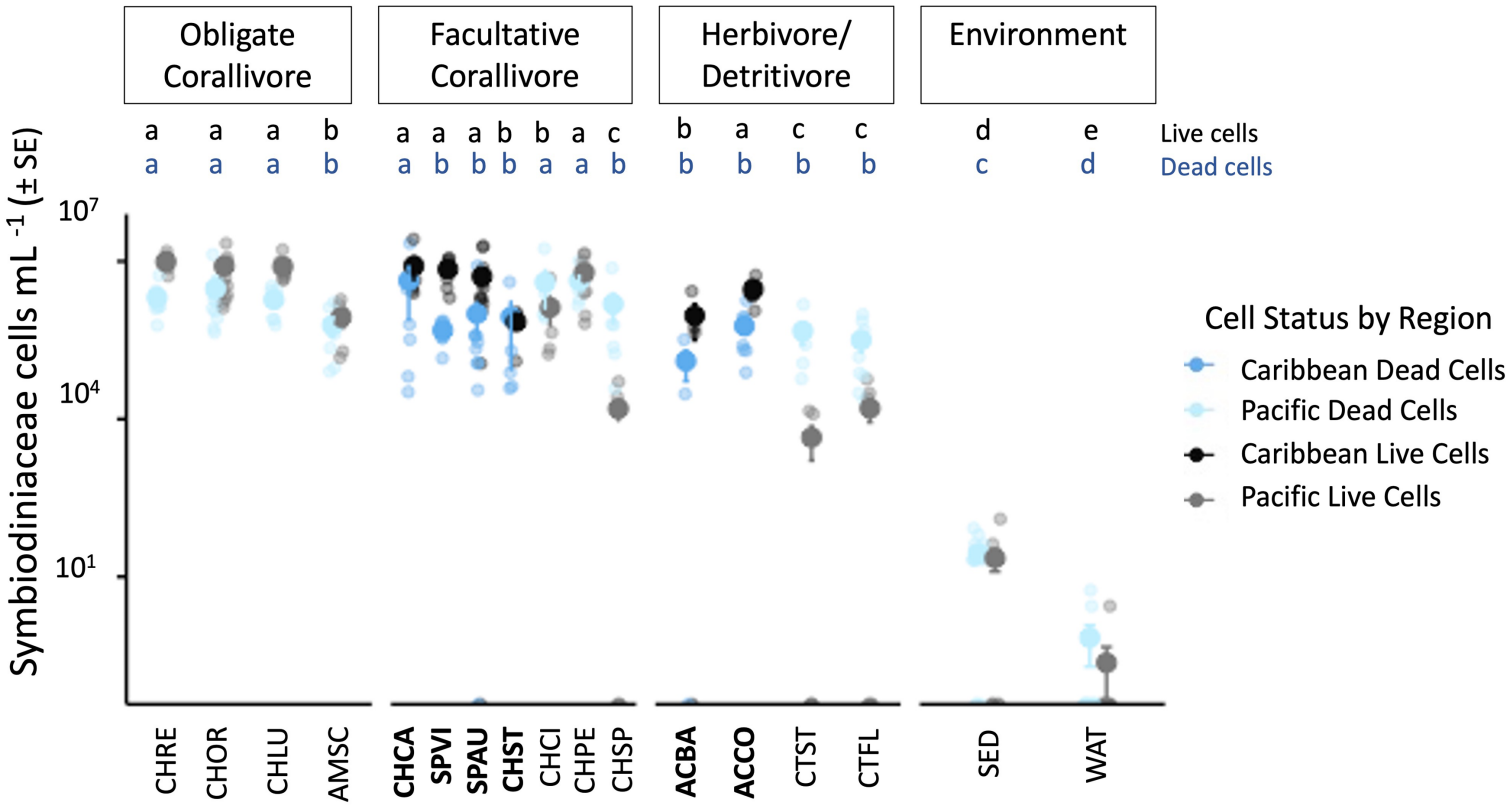
Total live and dead *Symbiodiniaceae* cell densities (cells per mL^−1^) across Caribbean and Pacific fish feces. Live cell densities are represented in black (Caribbean) and gray (Pacific), whereas dead cell densities are shown in dark blue (Caribbean) and light blue (Pacific). Pairwise PERMANOVA significant results for live cell densities are denoted with black letters a–e; dead cell densities are denoted with blue letters a–d. Caribbean fish species codes are in bold; Pacific fish and environmental codes are not. **ACBA**, *Acanthurus bahianus*; **ACCO**, *Acanthurus coeruleus*; AMSC, *Amanses scopas*; **CHCA**, *Chaetodon capistratus*; CHCI, *Chaetodon citrinellus*; CHLU, *Chaetodon lunulatus*; CHOR, *Chaetodon ornatissimus*; CHPE, *Chaetodon pelewensis*; CHRE, *Chaetodon reticulatus*; CHSP, *Cholurus spilurus*; **CHST**, *Chaetodon striatus*; CTFL, *Ctenochaetus flavicauda*; CTST, *Ctenochaetus striatus*; **SPAU**, *Sparisoma aurofrenatum*; **SPVI**, *Sparisoma viride*; SED, Pacific reef sediment; WAT, Pacific reef water.

The feces of Caribbean fishes contained high numbers of Symbiodiniaceae cells, with an average live cell density of 4,702,719 (SD ± 5,690,204) and average dead cell density of 1,271,629 (SD ± 3,592,431; Figure 2, Supplementary Table 6). Caribbean fish feces harbored significantly more live than dead Symbiodiniaceae cells per ml (Wilcoxon rank sum test: *W* = 795, *p* < 0.001). Live Symbiodiniaceae cell densities were not significantly different among Caribbean facultative corallivores (*M* = 5,612,835) and herbivore/detritivore fish feces (*M* = 2,503,274; Kruskal–Wallis test: *X*^2^ = 0.7889, df = 1, *p* = 0.3744) nor across fish families (Kruskal–Wallis test: *X*^2^ = 2.5688, df = 1, *p* = 0.2768; Figure 2). *C. capistratus* had the highest average of live cells ml^−1^ (*M* = 8,228,097 ± 9,324,969) and *Chaetodon striatus* had the lowest average of live cells ml^−1^ (*M* = 706,125 ± 372,988); there was a significant effect of fish species on live Symbiodiniaceae cell densities in feces (Kruskal–Wallis test: *X*^2^ = 16.9866, df = 5, *p* = 0.0045; Figure 2, Supplementary Table 7).

For dead Symbiodiniaceae cell densities, there were no significant differences across foraging groups (Kruskal–Wallis test: *X*^2^ = 0.0993, df = 1, *p* = 0.7526), fish families (Kruskal–Wallis test: *X*^2^ = 1.1558, df = 2, *p* = 0.5611) or fish species (Kruskal–Wallis test: *X*^2^ = 5.1765, df = 5, *p* = 0.3947; Supplementary Table 7). No effects of factors such as island, site, fish phase, fish size, or fish weight were detected on live or dead Symbiodiniaceae cell densities in feces (Supplementary Table 8).

### Live Symbiodiniaceae densities were higher in Caribbean fish trophic groups, relative to feces from Pacific trophic counterparts

Live Symbiodiniaceae cell densities in Caribbean fish feces ranged from 114,000 to 26,425,500 cells ml^−1^ (*M* = 4,702,719 ± 5,690,204), whereas live cell densities in Pacific feces ranged from 0 to 30,474,138 cells ml^−1^ (*M* = 3,492,144 ± 5,487,599, from Grupstra et al., 2021; Figure 2). Overall there was a significant difference between the live Symbiodiniaceae cell densities across region and foraging groups (Kruskal–Wallis test: *X*^2^ = 88.5734, df = 5, *p* < 0.001; Supplementary Table 9). Caribbean facultative corallivores had significantly higher live Symbiodiniaceae cell densities in their feces, relative to the feces of Pacific facultative corallivores (Dunn’s test: *Z* = 2.3491, *p* = 0.014) and Pacific herbivore/detritivores (Dunn’s test: *Z* = 5.0124, *p* < 0.001; Supplementary Table 9). Caribbean herbivore/detritivore feces had significantly higher live Symbiodiniaceae cell densities relative to Pacific herbivore/detritivore feces (Dunn’s test: *Z* = 3.5077, *p* < 0.001). Both Caribbean facultative corallivores (Dunn’s test: *Z* = 6.4159, *p* < 0.001) and Caribbean herbivores/detritivores (Dunn’s test: *Z* = 4.2968, *p* < 0.001) had higher live Symbiodiniaceae cell densities than water and sediment. No other comparisons of live Symbiodiniaceae cell densities were significantly different among the feces of Caribbean versus Pacific fish trophic groups.

### Caribbean fish feces contained fewer dead Symbiodiniaceae cells, relative to feces of Pacific reef fish

Dead Symbiodiniaceae cell densities in Caribbean fish feces ranged from 30,010 to 21,895,815 cells ml^−1^ (*M* = 1,271,629 ± 3,592,431), whereas dead Symbiodiniaceae cell densities in Pacific feces ranged from 0 to 17,457,000 cells ml^−1^ (*M* = 1,601,544 ± 2,653,361, from Grupstra et al., 2021; Figure 2). Overall there was a significant difference between the dead Symbiodiniaceae cell densities across region and foraging groups (Kruskal–Wallis test: *X*^2^ = 84.9118, df = 5, *p* < 0.001; Supplementary Table 9). Dead Symbiodiniaceae cell densities were significantly higher in Pacific obligate corallivore feces, than in the feces of Caribbean facultative corallivores (Dunn’s test: *Z* = −3.2553, *p* = 0.0012) or Caribbean herbivores/detritivores (Dunn’s test: *Z* = −2.8388, *p* = 0.0034; Supplementary Table 9). Dead Symbiodiniaceae cell densities were also higher in the feces of Pacific facultative corallivores than in the feces of Caribbean facultative corallivores (Dunn’s test: *Z* = −2.9900, *p* = 0.0023) or Caribbean herbivores/detritivores (Dunn’s test: *Z* = −2.7469, *p* = 0.0041). Feces of Pacific herbivore/detritivore fishes had similar dead Symbiodiniaceae cell densities to those in feces of Caribbean facultative corallivores and herbivores/detritivores. However, dead Symbiodiniaceae cell densities in Pacific environmental samples (water, sediments) were lower than those in feces of Caribbean facultative corallivores (Dunn’s test: *Z* = 4.8799, *p* < 0.001) and Caribbean herbivores/detritivores (Dunn’s test: *Z* = 3.4117, *p* < 0.001).

### Caribbean fish feces contain diverse Symbiodiniaceae assemblages that are also found in Caribbean hard corals

**Figure 3:**
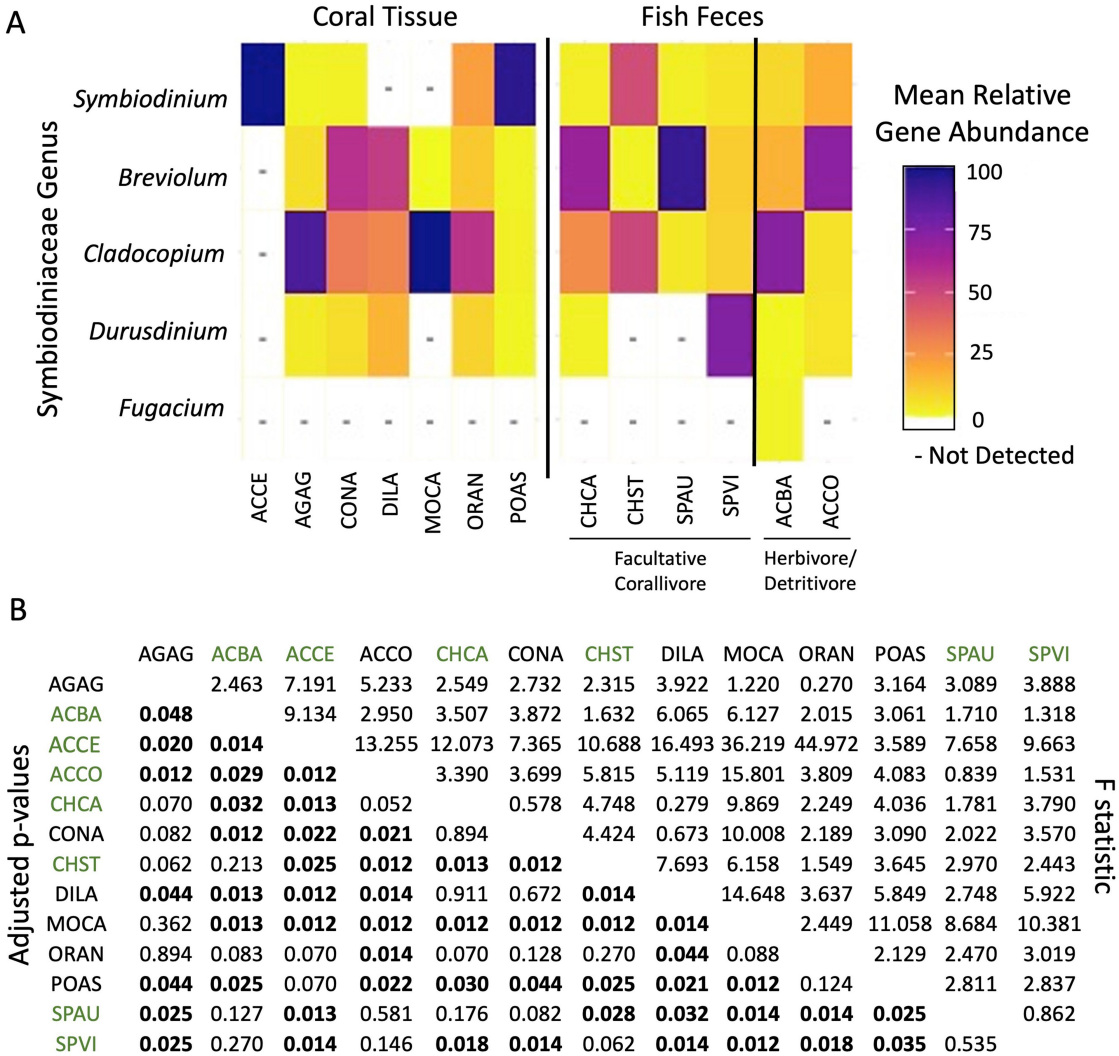
**(A)** Mean relative abundances of hits to five genera of Symbiodiniaceae (based on the internal transcribed spacer-2 (ITS-2) region of rDNA) in the tissues of seven species of Caribbean coral tissue, feces from four facultative corallivore fishes, and feces from two herbivore/detritivore fishes reveal intra- and inter-specific guild differences. All included samples had >200 reads. **(B)** Results from a pairwise PERMANOVA assessing the Symbiodiniaceae assemblages in Caribbean fish feces (green species codes) and Symbiodiniaceae communities in coral tissue (black species codes). Statistically significant adjusted *p*-values are in bold on the bottom left. *F*-statistics are on the top right. ACBA, *Acanthurus bahianus*; ACCE, *Acropora cervicornis*; ACCO, *Acanthurus coeruleus*; AGAG, *Agaricia agaricites*; CHCA, *Chaetodon capistratus*; CHST, *Chaetodon striatus*; CONA, *Colpophyllia natans*; DILA, *Diploria labyrinthiformis*; MOCA, *Montastraea cavernosa*; ORAN, *Orbicella annularis*; POAS, *Porites astreoides*; SPAU, *Sparisoma aurofrenatum*; SPVI, *Sparisoma viride*.

A total of five Symbiodiniaceae genera and 65 dominant ITS-2 type profiles (see Supplementary Table 1 for all 121 ITS-2 type profiles) were detected in Caribbean coral tissue and fish feces samples: 15 dominant ITS-2 DIVs were shared between these sample types, 28 dominant DIVs were detected in fish feces only, and 21 dominant DIVs were detected in coral only. *Breviolum* and *Cladocopium*, followed by *Symbiodinium*, were the most prevalent Symbiodiniaceae genera across all Caribbean samples (Figure 3, Table 1). Overall, the Symbiodiniaceae ITS-2 sequence assemblages in Caribbean stony coral tissues were significantly different from those in Caribbean fish feces (PERMANOVA: *F* = 28.298, df = 7, *p* < 0.001; Supplementary Figure 3B), but Symbiodiniaceae assemblages in some coral species and the feces of some fish species were not distinguishable statistically (Figure 3).

**Table 1:** The total number of samples (feces of Caribbean fish or Caribbean corals) containing a given *Symbiodiniaceae* internal transcribed spacer-2 (ITS-2) region dominant defining intragenomic variant (DIV, see Supplementary Table 1).

Dominant DIV	Found in		# of fish feces samples						# of coral samples						
	Fish feces	Coral	ACBA	ACCO	CHCA	CHST	SPAU	SPVI	ACCE	AGAG	CONA	DILA	MOCA	ORAN	POAS
A1dh	Yes	No	–	2	–	–	1	–	–	–	–	–	–	–	–
A2l	Yes	No	1	–	–	–	1	–	–	–	–	–	–	–	–
A2w	Yes	No	–	–	–	–	1	–	–	–	–	–	–	–	–
**A3**	**Yes**	**Yes**	**3**	**1**	**1**	**–**	**2**	**–**	**5**	**1**	**–**	**–**	**–**	**9**	**–**
A3/A4	Yes	No	–	3	–	–	1	1	–	–	–	–	–	–	–
A3/A4/A4a	Yes	No	–	–	3	–	–	1	–	–	–	–	–	–	–
**A4**	**Yes**	**Yes**	**3**	**1**	**–**	**2**	**1**	**1**	**–**	**–**	**–**	**–**	**–**	**–**	**1**
A4/A4a	No	Yes	–	–	–	–	–	–	–	–	–	–	–	–	19
A4/A4a/A4bz	No	Yes	–	–	–	–	–	–	–	–	–	–	–	–	9
A4/A4a/A4cb	No	Yes	–	–	–	–	–	–	–	1	–	–	–	–	6
A4/A4ea	No	Yes	–	–	–	–	–	–	–	–	–	–	–	–	9
A4cz	Yes	No	1	1	–	–	–	–	–	–	–	–	–	–	–
**A4df**	**Yes**	**Yes**	**2**	**2**	**–**	**–**	**–**	**1**	**–**	**–**	**–**	**–**	**–**	**1**	**–**
A4z	Yes	No	–	–	–	–	–	2	–	–	–	–	–	–	–
**A13**	**Yes**	**Yes**	**1**	**1**	**3**	**–**	**–**	**–**	**–**	**–**	**2**	**–**	**–**	**2**	**–**
**B1**	**Yes**	**Yes**	**1**	**6**	**3**	**1**	**3**	**1**	**–**	**1**	**5**	**17**	**1**	**9**	**2**
**B1/B5**	**Yes**	**Yes**	**4**	**1**	**–**	**–**	**2**	**1**	**–**	**–**	**3**	**–**	**–**	**–**	**–**
B1/B1do	Yes	No	–	–	2	–	1	–	–	–	–	–	–	–	–
B1/B19	Yes	No	1	–	4	–	–	–	–	–	–	–	–	–	–
B2/B1	Yes	No	–	–	1	–	–	–	–	–	–	–	–	–	–
B2d	Yes	No	1	–	–	–	–	–	–	–	–	–	–	–	–
B5	Yes	No	1	1	–	–	1	–	–	–	–	–	–	–	–
B7	Yes	No	2	1	3	–	–	1	–	–	–	–	–	–	–
B8a	Yes	No	–	3	1	–	–	–	–	–	–	–	–	–	–
B18e	Yes	No	2	–	–	–	1	1	–	–	–	–	–	–	–
B18e/B7/B1	Yes	No	1	1	–	–	1	3	–	–	–	–	–	–	–
**B19**	**Yes**	**Yes**	**2**	**–**	**1**	**–**	**–**	**–**	**–**	**1**	**23**	**–**	**–**	**–**	
B19/B5af	No	Yes	–	–	–	–	–	–	–	–	8	–	–	–	–
B19bb	Yes	No	2	–	5	–	–	–	–	–	–	–	–	–	–
B19bc	No	Yes	–	–	–	–	–	–	–	–	3	–	–	–	–
C1	**Yes**	**Yes**	**3**	**1**	**4**	**4**	**1**	**1**	**–**	**–**	**2**	**–**	**–**	**–**	**–**
C1/C1c	**Yes**	**Yes**	**–**	**–**	**–**	**–**	**1**	**–**	**–**	**–**	**1**	**1**	**–**	**–**	**3**
C1/C1c/C3	**Yes**	**Yes**	**–**	**–**	**1**	**1**	**–**	**–**	**–**	**1**	**–**	**–**	**–**	**–**	**–**
C1/C3	Yes	No	–	–	–	–	1	2	–	–	–	–	–	–	–
C1/C7	Yes	No	–	–	2	–	–	–	–	–	–	–	–	–	–
C1/C42.2	Yes	No	–	2	–	–	–	–	–	–	–	–	–	–	–
C1/C42.2/C1b/C3	No	Yes	–	–	–	–	–	–	–	–	–	–	–	–	2
C1/C42.2/C3/C1c	Yes	No	–	–	–	–	–	–	–	–	–	–	–	–	–
C3	No	Yes	–	–	–	–	–	–	–	2	–	–	39	1	1
**C3/C3b**	**Yes**	**Yes**	**–**	**–**	**3**	**–**	**–**	**–**	**–**	**22**	**–**	**–**	**–**	**–**	**4**
C3/C3fe	No	No	–	–	–	–	–	–	–	–	–	–	–	–	–
C3/C3u	No	Yes	–	–	–	–	–	–	–	–	–	–	–	–	1
C3b	No	Yes	–	–	–	–	–	–	–	11	–	–	–	–	–
**C3b/C3/C3abc**	**Yes**	**Yes**	**–**	**–**	**–**	**–**	**–**	**1**	**–**	**4**	**–**	**1**	**–**	**–**	**–**
C3b/C3fe	No	Yes	–	–	–	–	–	–	–	–	–	–	–	–	1
C3fe	No	Yes	–	–	–	–	–	–	–	–	17	5	–	–	1
C3u	No	Yes	–	–	–	–	–	–	–	1	–	–	–	–	–
C7	Yes	No	–	–	–	–	–	1	–	–	–	–	–	–	–
C7/C12c/C7d	No	Yes	–	–	–	–	–	–	–	1	–	–	–	15	1
C7/C3	No	Yes	–	–	–	–	–	–	–	–	–	–	–	–	1
C12c/C7	No	Yes	–	–	–	–	–	–	–	–	–	–	–	3	–
C12c/C7/C7d	No	Yes	–	–	–	–	–	–	–	–	–	–	–	9	1
C21/C1	No	Yes	–	–	–	–	–	–	–	–	–	–	–	–	1
C42.2/C45a	Yes	No	–	–	2	–	–	–	–	–	–	–	–	–	–
C44	Yes	No	–	–	–	–	–	1	–	–	–	–	–	–	–
C54b/C54a	No	Yes	–	–	–	–	–	–	–	–	–	2	–	–	–
**C66**	**Yes**	**Yes**	**–**	**–**	**–**	**–**	**–**	**1**	**–**	**–**	**–**	**–**	**–**	**–**	**1**
C80	No	Yes	–	–	–	–	–	–	–	–	–	–	–	–	2
C80b	No	Yes	–	–	–	–	–	–	–	–	–	–	–	–	2
C91	Yes	No	–	–	–	–	1	–	–	–	–	–	–	–	–
**D1**	**Yes**	**Yes**	**–**	**2**	**1**	**–**	**–**	**1**	**–**	**1**	**4**	**9**	**–**	**5**	**1**
**D1/D4/D2**	**Yes**	**Yes**	**1**	**2**	**1**	**–**	**–**	**–**	**–**	**1**	**3**	**–**	**–**	**2**	**–**
D1/D4/D4c	Yes	No	–	–	–	–	–	1	–	–	–	–	–	–	–
F5.1	Yes	No	2	–	–	–	–	–	–	–	–	–	–	–	–
F5bf	Yes	No	2	–	–	–	–	–	–	–	–	–	–	–	–
**Total dominant DIVs**	**43**	**36**	**20**	**17**	**18**	**4**	**16**	**8**	**1**	**13**	**11**	**6**	**2**	**10**	**21**

The bottom row of the table denotes the total number of dominant DIVs documented from a given species. *Symbiodiniaceae* ITS-2 dominant DIVs recovered from both coral and fish feces are in bold. ACBA, *Acanthurus bahianus*; ACCE, *Acropora cervicornis*; ACCO, *Acanthurus coeruleus*; AGAG, *Agaricia agaricites*; CHCA, *Chaetodon capistratus*; CHST, *Chaetodon striatus*; CONA, *Colpophyllia natans*; DILA, *Diploria labyrinthiformis*; MOCA, *Montastraea cavernosa*; ORAN, *Orbicella annularis*; POAS, *Porites astreoides*; SPAU, *Sparisoma aurofrenatum*; SPVI, *Sparisoma viride*.

Four Symbiodiniaceae genera (listed from most to least frequently detected: *Cladocopium*, *Symbiodinium*, *Breviolum*, and *Durusdinium*) and 36 dominant ITS-2 DIVs were recovered from Caribbean corals (total *n* = 244, Figure 3, Table 1). Individual coral species harbored from 1 to 4 Symbiodiniaceae genera (with total number of type profiles: *A. cervicornis -* 1; *A. agaricites* - 13; *C. natans* - 11; *D. labyrinthiformis* - 6; *M. cavernosa -* 2*; O. annularis* - 10; *P. astreoides* - 21; Figure 3, Table 1). The 21 dominant ITS-2 DIVs detected from corals only were rare, each appearing in 1 or 2 samples only (except for *P. astreoides*; Table 1).

Across feces from six species of Caribbean fish (total *n* = 46, Supplementary Figure 1), five Symbiodiniaceae genera (listed from most frequently detected to least frequent: *Breviolum*, *Cladocopium*, *Symbiodinium*, *Durusdinium*, and *Fugacium*) and 43 dominant ITS-2 DIVs were detected (Figure 3, Table 1). Individual fish fecal assemblages ranged from containing 3–5 Symbiodiniaceae genera (with total number of type profiles: *A. bahianus* - 20; *A. coeruleus* - 17; *C. capistratus* - 18; *C. striatus* - 4; *S. aurofrenatum* - 16; *S. viride* - 18; Figure 3, Table 1). The 28 dominant ITS-2 DIVs detected from fish feces only were relatively abundant across samples (Table 1).

Symbiodiniaceae ITS-2 sequence assemblages differed across Caribbean coral species (PERMANOVA: df = 6, *F* = 4.958, *p* < 0.001, Supplementary Figure 3). Symbiodiniaceae ITS-2 sequence assemblages in Caribbean fish feces also differed at the species-level (PERMANOVA: df = 5, *F* = 2.617, *p* < 0.001), but no differences were detected among foraging groups (PERMANOVA: df = 1, *F* = 1.543, *p* = 0.158). Butterflyfish feces drove these species-level differences; a pairwise PERMANOVA revealed significant differences in Symbiodiniaceae compositions between *C. capistratus* and *A. bahianus* (Pairwise: df = 1.17, *F* = 3.5067, *p* = 0.032; Figure 3), *S. viride* (Pairwise: df = 1.15, *F* = 3.7904, *p* = 0.018), and *Chaetodon striatus* (Pairwise: df = 1.14, *F* = 4.7485, *p* = 0.013). Additionally, Symbiodiniaceae ITS-2 sequence assemblages in *Chaetodon striatus* feces differed from those in *A. coeruleus* (Pairwise: df = 1.17, *F* = 5.8149, *p* = 0.012) and *S. aurofrenatum* (Pairwise: df = 1.17, *F* = 2.9701, *p* = 0.028). As expected, Caribbean and Pacific Symbiodiniaceae assemblages were different (Supplementary Figure 4, Supplementary Table 10).

## Discussion

This study demonstrates that Caribbean fish feces are a substantial environmental pool of relevant dinoflagellate symbionts for corals. We documented that the Symbiodiniaceae genera and dominant ITS-2 DIVs present in the feces of six Caribbean fish species overlaps substantially with the symbiont communities found in the coral species they consume (Figure 3, Table 1). Fish feces and corals shared 15 dominant ITS-2 DIVs, including ITS-2 type profiles common in Caribbean corals (e.g., B1, C3/C21, D1/D2/D4, Santos et al., 2004; Finney et al., 2010; Baker et al., 2016; Teschima et al., 2019; Nitschke et al., 2022; Million et al., 2025). For example, we observed *C. capistratus* feeding on *A. agaricites*, *C. natans*, *D. labyrinthiformis*, *O. annularis*, and *P. astreoides* and documented that the feces of this fish shared nine dominant DIVs with the corals they consumed (Table 1). The feces of the six fish species also contained high densities of live Symbiodiniaceae cells (~114,000 to 26 million cells ml^−1^; Figure 2)—orders of magnitude more than seawater (~10 to 10^1^ cells ml^−1^), sediments (10^1^–10^3^ cells ml^−1^), and macroalgae surfaces (10^2^–10^3^ cells ml^−1^; references summarized therein; Fujise et al., 2021; Grupstra et al., 2021; Bell and Quigley, 2025). Overall, our findings from USVI reefs add to mounting evidence that fish feces are an environmental hotspot of microbial symbionts relevant to corals globally (Muller Parker, 1984; Grupstra et al., 2021; Castro-Sanguino and Sánchez, 2012; Grupstra et al., 2023; Clever et al., 2022). We posit that trophic transmission of Symbiodiniaceae by these fishes to prospective coral hosts is likely, through direct contact between corals and feces and/or through disintegration of feces, releasing Symbiodiniaceae cells into other environmental pools (e.g., sediments and macroalgal surfaces).

### Live Symbiodiniaceae densities are consistently high in the feces of Caribbean fish across foraging groups, in contrast to more variable patterns in Pacific fish feces

This study revealed that live Symbiodiniaceae cell densities in the feces of Caribbean facultative corallivores and herbivores/detritivores were equivalent (and comparable to densities in Pacific obligate corallivore feces, Grupstra et al., 2021; Figure 2, Supplementary Table 9). This highlights a regional difference with Pacific reefs, where obligate corallivores had higher densities of Symbiodiniaceae in their feces, relative to herbivores/detritivores (Supplementary Tables 2, 3). Ratios of live to dead Symbiodiniaceae cells in fish feces also differed regionally, with Caribbean herbivore/detritivore feces having more live cells than dead (this study), unlike some Pacific facultative corallivores and all sampled herbivore/detritivore feces (Grupstra et al., 2021). Two stressors, SCTLD in the Caribbean and bleaching in the Pacific, as well as aspects of fish biology and behavior, likely explain regional differences in Symbiodiniaceae density in fish feces.

In 2022, during sampling, USVI reefs were in the midst of the endemic phase of a SCTLD outbreak (Brandt et al., 2021; Swaminathan et al., 2024), likely leading to an increase in sloughed coral tissues within the water column, sediments, and on other surfaces that created incidental opportunities for herbivores/detritivores to ingest symbiont cells during foraging. Degradation of Symbiodiniaceae cells is a notable feature of SCTLD pathology (McDonald, 2020; Beavers et al., 2023; Evans et al., 2023; Rossin et al., *in review*) and likely contributed to observed patterns in live versus dead Symbiodiniaceae cell densities in Caribbean fish feces analyzed in this study. A severe coral bleaching event (Leinbach et al., 2021; Speare et al., 2022) was ongoing on Mo’orean reefs (South Pacific) during sampling by Grupstra et al. (2021) likely increasing the proportion of degraded symbiont cells in coral tissues, potentially altering the ratio of live to dead cells detected in fish feces (Howe-Kerr et al., 2023a,b, Fujise et al., 2013). Although USVI (this study) and Mo’orean (Grupstra et al., 2021) reefs each experienced significant stress events during sampling, it is unlikely that these events completely explain the regional differences quantified in this study. Monitoring of Symbiodiniaceae live and dead cell densities in the feces of Caribbean and Pacific fish during additional stress events, as well as during ambient periods, is necessary to further resolve the dynamics of Symbiodiniaceae transfer from corals to environmental pools via consumer feeding.

### Diet, anatomical, and digestive differences may explain similar Symbiodiniaceae densities in the feces of USVI reef fishes

It was surprising to find similar densities of Symbiodiniaceae in the feces of facultative corallivores and herbivore/detritivores on USVI reefs. A combination of factors likely contributed to this, including dietary shifts, as well as anatomical and digestive differences among trophic groups. Low average coral cover (~12%) on USVI reefs may have minimized dietary distinctions that originally evolved between facultative corallivores and herbivorous/detritivorous fishes during periods of higher coral cover (Floeter et al., 2004; Cole et al., 2008; Mouillot et al., 2014; Titus et al., 2019). Most Caribbean facultative corallivores in this study, like herbivores/detritivores, primarily consumed macroalgae or rock/sand substrates, biting stony corals only 16% of the time (366 of 2,233 total bites). *Chaetodon capistratus* was an exception for the facultative corallivores, with hard coral comprising approximately 60% of its diet (318 of 528 total bites recorded; Figure 1; Supplementary Table 2). Yet, despite a higher proportion of coral bites, *C. capistratus* fed less frequently (528 bites recorded) than other facultative corallivores (parrotfish: average 822 bites recorded) and herbivore/detritivores (average of 1,064 total bites). This likely explains why Symbiodiniaceae densities in *C. capistratus* feces were not significantly different from those of other Caribbean fish sampled here. Reef fishes, including surgeonfishes, have been documented exhibiting opportunistic foraging behavior (Delgado-Pech et al., 2020), potentially consuming coral tissue directly or feeding on substrates that harbor free-living Symbiodiniaceae (Tremblay et al., 2016; Winkler et al., 2015). In this study, surgeonfishes, which are typically categorized as herbivores/detritivores, were observed foraging on corals (60 total coral bites out of 2,128 total bites); this likely explains the Symbiodiniaceae densities observed in their feces.

The presence of diseased coral tissue could have potentially altered foraging patterns, with implications for Symbiodiniaceae densities in sampled fish feces. In the Florida Keys (U.S.A.), *C. capistratus* was observed to preferentially forage on SCTLD-affected coral tissue (Noonan and Childress, 2020; Titus et al., 2022). In our fish foraging observations on USVI reefs, 20% of fish bites (75 bites on diseased corals out of 378 total bites on stony corals; Figure 1, Supplementary Table 2) were on diseased corals. Given the low prevalence of disease observed across USVI study sites, the observed foraging behavior suggests a preference for diseased coral in at least *C. capistratus*, which accounted for 89% of the total bites from diseased corals (67 of 75 total bites from diseased corals). We interpret that this is why *C. capistratus* had significantly higher densities of dead Symbiodiniaceae in its feces, relative to other Caribbean fishes (Figure 2).

Beyond observed bites, differences in jaw gape size and/or digestive mechanisms among butterflyfish, parrotfish, and surgeonfish likely contributed to the equivalent densities of Symbiodiniaceae found in feces across Caribbean trophic groups (Wainwright and Price, 2018; Barott et al., 2021; Schiettekatte et al., 2023). For example, members of the family Chaetodontidae possess an intermandibular joint that enables them to scrape coral surfaces (Konow and Ferry, 2013). In contrast, Scarinae have an intramandibular joint, are generally larger (with larger gape sizes) with elongated mandibular bones, have robust adductor muscles, and specialized cutting dentition, which allow them to excavate deeper into coral substrates (Rotjan and Lewis, 2008; Wainwright and Price, 2018). These anatomical adaptations likely enable parrotfish to remove greater volumes of coral tissue per bite (containing more Symbiodiniaceae cells) compared to butterflyfish, which take more superficial bites. Thus, although parrotfish took fewer bites on corals than *C. capistratus* in this study, they likely consumed relatively more Symbiodiniaceae cells per bite. Similarly, nutrient assimilation varies across trophic guilds, with absorption and egestion rates (Ghilardi et al., 2021; Schiettekatte et al., 2023), as well as gut lengths and digestive strategies (mechanical versus chemical) differing between corallivores and herbivores (Clements et al., 2009; German et al., 2015). Future studies should comparatively track the condition of individual Symbiodiniaceae cells through the guts of fish species with different gut lengths and digestive strategies.

### Diverse Symbiodiniaceae assemblages in Caribbean fish feces are consistent with generalist fish foraging behavior

This study detected all Symbiodiniaceae genera that typically associate with stony corals (*Symbiodinium*, *Breviolum*, *Cladocopium*, and *Durusdinium*) in Caribbean fish feces, as well as *Fugacium* (Figure 3). The diversity of Symbiodiniaceae assemblages in Caribbean fish feces was overall higher than that in Pacific fish feces (Grupstra et al., 2021; Supplementary Figure 4; Supplementary Table 10). This pattern aligns with expectations based on differences in the biogeographic distributions of dominant Symbiodiniaceae lineages between the Caribbean and Pacific regions (Baker et al., 2003; LaJeunesse et al., 2003). Although coral-Symbiodiniaceae associations are overall non-random and host-selective (Davies et al., 2023), corals in this study frequently harbored multiple symbiont lineages (Figure 3, Table 1; Baker and Romanski, 2007; Silverstein et al., 2012). Overall, the generalist foraging behaviors of these fishes likely contribute to the presence of composite fecal assemblages representing Symbiodiniaceae from a broad array of hard coral, soft coral, and macroalgae.

Many Caribbean reef fish, including the six species examined here, exhibit generalist foraging behavior within their trophic groups (Bruggemann et al., 1994; McAfee, 1994; Bellwood et al., 2010; Vlam, 2024). Despite low coral cover, USVI facultative corallivores and some herbivores/detritivores were observed feeding on multiple coral species (in addition to non-coral substrates; Figure 1; Supplementary Table 2). Our fish follows, combined with amplicon sequencing of Symbiodiniaceae ITS-2 rDNA from fish feces and coral tissues, support the conclusion that these fishes may feed on a range of coral taxa (where available) and then redistribute the Symbiodiniaceae of these corals in their feces. For example, as mentioned above surgeonfishes are categorized as herbivores/detritivores, yet they were observed foraging on a range of coral species – *O. annularis*, *O. franksi*, *M. cavernosa*, *P. porites* and *Gorgonia* sp.– and their feces contained diverse Symbiodiniaceae assemblages that closely matched the symbiont communities of corals sampled in this study and those previously reported in the literature (Figure 3, Table 1).

Fecal Symbiodiniaceae assemblages exhibited overlap with the Symbiodiniaceae diversity associated with coral species sampled in this study (Figure 3, Table 1). However, fish were also observed foraging on coral species not included in our tissue sampling, including *Orbicella franksi*, *Siderastrea siderea*, *Porites porites*, *Gorgonia* sp. These corals are known to associate with Symbiodiniaceae ITS-2 lineages such as *Breviolum* B1, *Cladocopium* C1, and *Durusdinium* D1a (Green et al., 2014; Davies et al., 2018; Terraneo et al., 2019; Shirur et al., 2014) – all of which were detected in the Caribbean fish feces sampled here (Figure 3, Table 1). In this study, 22 Symbiodiniaceae ITS-2 type profiles were identified in coral tissues but not in feces. These type profiles were rare, appearing in only one or two corals sampled and thus may be less likely to appear in fish feces.

### Conclusion

This study provides the first quantification of Symbiodiniaceae density and diversity in Caribbean reef fish feces across multiple trophic groups. We show that fish feces represent a major reservoir of viable Symbiodiniaceae, with densities exceeding other environmental pools by several orders of magnitude. Symbiodiniaceae diversity (genera, dominant DIVs) in fish feces overlaps with that in local stony corals, indicating that fish likely redistribute coral-associated symbionts during foraging activities. Across trophic groups, Caribbean facultative corallivores and herbivores/detritivores contained equivalent, consistently high densities of live symbionts, challenging the assumption that diet governs Symbiodiniaceae in feces. Instead, low coral cover, generalist foraging, and anatomical and digestive traits likely diminish trophic distinctions in feces among fish trophic groups. Regional stress events such as SCTLD and bleaching may further modulate the density and viability of Symbiodiniaceae in consumer feces; further examination across disturbed and ambient conditions is needed.

Importantly, previous work has demonstrated that consumer feces can accelerate symbiont acquisition by cnidarians, suggesting that trophic transmission of Symbiodiniaceae could contribute to host recovery and resilience following heat stress. Our findings suggest that reef fishes can serve as vectors facilitating the redistribution of viable symbionts across reef habitats. Consequently, reducing fishing pressure on Caribbean reefs may indirectly enhance coral recovery, particularly during periods of environmental disturbance. Moreover, these trophic transmission benefits extend beyond corallivorous species: herbivore/detritivores and other trophic groups, as well as non-fish consumers, can contribute to reef connectivity and ecosystem resilience by distributing viable symbionts of benthic organisms. Future studies should explore the extent to which consumer–symbiont pathways can be leveraged or replicated to support coral persistence and inform reef restoration strategies under ongoing global change.

## Data Availability

The data presented in this study are publicly available. The data can be found here: https://www.ncbi.nlm.nih.gov/bioproject, accession PRJNA1390826.

## References

[ref1] Ali A. Kriefall N. G. Emery L. E. Kenkel C. D. Matz M. V. Davies S. W. (2019). Recruit symbiosis establishment and Symbiodiniaceae composition influenced by adult corals and reef sediment. Coral Reefs 38, 405–415.

[ref2] Anderson M. J. (2006). Distance-based tests for homogeneity of multivariate dispersions. Biometrics 62, 245–253. 16542252 10.1111/j.1541-0420.2005.00440.x

[ref3] Anderson M. J. Walsh D. C. I. (2013). PERMANOVA, ANOSIM, and the mantel test in the face of heterogeneous dispersions: what null hypothesis are you testing? Ecol. Monogr. 83, 557–574. doi: 10.1890/12-2010.1

[ref4] Baker A. C. (2003). Flexibility and specificity in coral-algal symbiosis: diversity, ecology, and biogeography of Symbiodinium. Annu. Rev. Ecol. Evol. Syst. 34, 661–689.

[ref5] Baker A. C. Correa A. M. Cunning R. (2016). “Diversity, distribution and stability of Symbiodinium in reef corals of the eastern tropical pacific” in Coral reefs of the eastern tropical Pacific: Persistence and loss in a dynamic environment (Dordrecht: Springer Netherlands), 405–420.

[ref6] Baker A. C. Romanski A. M. (2007). Multiple symbiotic partnerships are common in scleractinian corals, but not in octocorals: comment on Goulet (2006). Mar. Ecol. Prog. Ser. 335, 237–242.

[ref7] Beavers K. M. Van Buren E. W. Rossin A. M. Emery M. A. Veglia A. J. Karrick C. E. . (2023). Stony coral tissue loss disease induces transcriptional signatures of in situ degradation of dysfunctional Symbiodiniaceae. Nat. Commun. 14:2915. 37217477 10.1038/s41467-023-38612-4 PMC10202950

[ref8] Bell S. L. Quigley K. M. (2025). Global free-living Symbiodiniaceae biodiversity mirrors local environments. J. Biogeogr. e15137.

[ref9] Bellwood D. Klanten S. Cowman P. Pratchett M. Konow N. Van Herwerden L. (2010). Evolutionary history of the butterflyfishes (f: Chaetodontidae) and the rise of coral feeding fishes. J. Evol. Biol. 23, 335–349. 20487131 10.1111/j.1420-9101.2009.01904.x

[ref10] Brandt M. E. Ennis R. S. Meiling S. S. Townsend J. Cobleigh K. Glahn A. . (2021). The emergence and initial impact of stony coral tissue loss disease (SCTLD) in the United States Virgin Islands. Front. Mar. Sci. 8:715329.

[ref11] Bright M. Bulgheresi S. (2010). A complex journey: transmission of microbial symbionts. Nat. Rev. Microbiol. 8, 218–230. 20157340 10.1038/nrmicro2262 PMC2967712

[ref12] Castro-Sanguino C. Sánchez J. A. (2012). Dispersal of Symbiodinium by the stoplight parrotfish *Sparisoma viride*. Biol. Lett. 8, 282–286. 21957090 10.1098/rsbl.2011.0836 PMC3297393

[ref13] Clements K. D. Raubenheimer D. Choat J. H. (2009). Nutritional ecology of marine herbivorous fishes: ten years on. Funct. Ecol. 23, 79–92.

[ref14] Clever F. Sourisse J. M. Preziosi R. F. Eisen J. A. Guerra E. C. R. Scott J. J. . (2022). The gut microbiome variability of a butterflyfish increases on severely degraded Caribbean reefs. Communications biology 5:770. 35908086 10.1038/s42003-022-03679-0 PMC9338936

[ref15] Cole A. J. Pratchett M. S. Jones G. P. (2008). Diversity and functional importance of coral-feeding fishes on tropical coral reefs. Fish Fish. 9, 286–307.

[ref16] Cunning R. Lenz E. A. Edmunds P. J. (2024). Measuring multi-year changes in the Symbiodiniaceae algae in Caribbean corals on coral-depleted reefs. PeerJ 12:e17358. 38827291 10.7717/peerj.17358 PMC11141555

[ref17] Davies S. W. Ries J. B. Marchetti A. Castillo K. D. (2018). Symbiodinium functional diversity in the coral *Siderastrea siderea* is influenced by thermal stress and reef environment, but not ocean acidification. Front. Mar. Sci. 5:150.

[ref18] Davies S. W. Gamache M. H. Howe-Kerr L. I. Kriefall N. G. Baker A. C. Banaszak A. T. . (2023). Building consensus around the assessment and interpretation of Symbiodiniaceae diversity. PeerJ 11:e15023. 37151292 10.7717/peerj.15023 PMC10162043

[ref19] Delgado-Pech B. Almazán-Becerril A. Peniche-Pérez J. Caballero-Vázquez J. A. (2020). Facultative scavenging feeding habits in *Acanthurus chirurgus* (Bloch, 1787)(Acanthuriformes: Acanthuridae). Biodiversity Data Journal 8:e53712. 32821209 10.3897/BDJ.8.e53712 PMC7398945

[ref20] Dinno A. Dinno M. A. (2017). Package ‘dunn. test’. CRAN Repos 10, 1–7.

[ref21] Evans J. S. Paul V. J. Ushijima B. Pitts K. A. Kellogg C. A. (2023). Investigating microbial size classes associated with the transmission of stony coral tissue loss disease (SCTLD). PeerJ 11:e15836. 37637172 10.7717/peerj.15836 PMC10460154

[ref22] Finney J. C. Pettay D. T. Sampayo E. M. Warner M. E. Oxenford H. A. LaJeunesse T. C. (2010). The relative significance of host–habitat, depth, and geography on the ecology, endemism, and speciation of coral endosymbionts in the genus Symbiodinium. Microb. Ecol. 60, 250–263. 20502891 10.1007/s00248-010-9681-y

[ref23] Floeter S. R. Ferreira C. E. L. Dominici-Arosemena A. Zalmon I. R. (2004). Latitudinal gradients in Atlantic reef fish communities: trophic structure and spatial use patterns. J. Fish Biol. 64, 1680–1699.

[ref24] Fujise L. Suggett D. J. Stat M. Kahlke T. Bunce M. Gardner S. G. . (2021). Unlocking the phylogenetic diversity, primary habitats, and abundances of free-living Symbiodiniaceae on a coral reef. Mol. Ecol. 30, 343–360. 33141992 10.1111/mec.15719

[ref25] Fujise L. Yamashita H. Suzuki G. Koike K. (2013). Expulsion of zooxanthellae (Symbiodinium) from several species of scleractinian corals: comparison under non-stress conditions and thermal stress conditions. Galaxea, Journal of Coral Reef Studies 15, 29–36.

[ref26] Garnier S. Ross N. Rudis R. Camargo P. Sciaini M. Scherer C. (2023). Viridis (Lite)-Colorblind-friendly color maps for R. Version 0:3.

[ref27] German D. P. Sung A. Jhaveri P. Agnihotri R. (2015). More than one way to be an herbivore: convergent evolution of herbivory using different digestive strategies in fishes. Zoology 118, 161–170. 25769813 10.1016/j.zool.2014.12.002

[ref28] Ghilardi M. Schiettekatte N. M. Casey J. M. Brandl S. J. Degregori S. Mercière A. . (2021). Phylogeny, body morphology, and trophic level shape intestinal traits in coral reef fishes. Ecol. Evol. 11, 13218–13231. 34646464 10.1002/ece3.8045 PMC8495780

[ref29] Green E. A. Davies S. W. Matz M. V. Medina M. (2014). Quantifying cryptic Symbiodinium diversity within Orbicella faveolata and Orbicella franksi at the flower garden banks. Gulf of Mexico. *PeerJ* 2:e386. 24883247 10.7717/peerj.386 PMC4034615

[ref30] Grupstra C. G. Howe-Kerr L. I. van der Meulen J. A. Veglia A. J. Coy S. R. Correa A. M. (2023). Consumer feces impact coral health in guild-specific ways. Front. Mar. Sci. 10:1110346.

[ref31] Grupstra C. G. Rabbitt K. M. Howe-Kerr L. I. Correa A. M. (2021). Fish predation on corals promotes the dispersal of coral symbionts. Animal Microbiome 3, 1–12. 33752761 10.1186/s42523-021-00086-4 PMC7986512

[ref32] Harmelin-Vivien M. L. (2002). “Energetics and fish diversity on coral reefs” in Coral reef fishes: Dynamics and diversity in a complex ecosystem. ed. Sale P. (Academic Press), 265–274.

[ref33] Harrison P. L. Wallace C. C. (1990). “Reproduction, dispersal and recruitment of scleractinian corals” in Coral reefs, vol. 25 (Amsterdam, The Netherlands: Elsevier Science Publishers), 133–207.

[ref34] Heine J. H. (2014). Pairwise: Rasch model parameters by pairwise algorithm. *Computer software*. Munich. Zugriff am 28:2017.

[ref35] Howe-Kerr L. I. Grupstra C. G. Rabbitt K. M. Conetta D. Coy S. R. Klinges J. G. . (2023). Viruses of a key coral symbiont exhibit temperature-driven productivity across a reefscape. ISME communications 3:27. 37009785 10.1038/s43705-023-00227-7 PMC10068613

[ref36] Howe-Kerr L. I. Knochel A. M. Meyer M. D. Sims J. A. Karrick C. E. Grupstra C. G. . (2023). Filamentous virus-like particles are present in coral dinoflagellates across genera and ocean basins. ISME J. 17, 2389–2402. 37907732 10.1038/s41396-023-01526-6 PMC10689786

[ref37] Howe-Kerr L. I. Bachelot B. Wright R. M. Kenkel C. D. Bay L. K. Correa A. M. (2020). Symbiont community diversity is more variable in corals that respond poorly to stress. Glob. Chang. Biol. 26, 2220–2234. 32048447 10.1111/gcb.14999

[ref38] Hume B. C. Smith E. G. Ziegler M. Warrington H. J. Burt J. A. LaJeunesse T. C. . (2019). SymPortal: a novel analytical framework and platform for coral algal symbiont next-generation sequencing ITS2 profiling. Mol. Ecol. Resour. 19, 1063–1080. 30740899 10.1111/1755-0998.13004 PMC6618109

[ref39] Hume B. C. Ziegler M. Poulain J. Pochon X. Romac S. Boissin E. . (2018). An improved primer set and amplification protocol with increased specificity and sensitivity targeting the Symbiodinium ITS2 region. PeerJ 6:e4816. 29844969 10.7717/peerj.4816 PMC5970565

[ref40] Kassambara A. (2023). *Rstatix*: pipe-friendly framework for basic statistical tests. R package version 0:2. https://rpkgs.datanovia.com/rstatix/

[ref41] Kenkel C. D. Bay L. K. (2018). Exploring mechanisms that affect coral cooperation: symbiont transmission mode, cell density and community composition. PeerJ 6:e6047. 30533318 10.7717/peerj.6047 PMC6282938

[ref42] Konow N. Ferry-Graham L. A. (2013). Functional morphology of butterflyfishes. The Biology of butterflyfishes, 19–47.

[ref43] LaJeunesse T. C. Loh W. K. Van Woesik R. Hoegh-Guldberg O. Schmidt G. W. Fitt W. K. (2003). Low symbiont diversity in southern great barrier reef corals, relative to those of the Caribbean. Limnol. Oceanogr. 48, 2046–2054.

[ref44] LaJeunesse T. C. Parkinson J. E. Gabrielson P. W. Jeong H. J. Reimer J. D. Voolstra C. R. . (2018). Systematic revision of Symbiodiniaceae highlights the antiquity and diversity of coral endosymbionts. Curr. Biol. 28, 2570–2580. e2576 30100341 10.1016/j.cub.2018.07.008

[ref45] Leinbach S. E. Speare K. E. Rossin A. M. . (2021). Energetic and reproductive costs of coral recovery in divergent bleaching responses. Sci. Rep. 11:23546. doi: 10.1038/s41598-021-02807-w 34876599 PMC8651640

[ref46] Littman R. A. Van Oppen M. J. H. Willis B. L. (2008). Methods for sampling free-living Symbiodinium (zooxanthellae) and their distribution and abundance at Lizard Island (great barrier reef). J Exp Mar Bio Ecol. 364, 48–53. doi: 10.1016/j.jembe.2008.06.034

[ref47] McAfee S. T. (1994). Resource use by five sympatric parrotfishes in the san Blas archipelago. Panama: San Francisco State University, San Francisco, CA, USA.

[ref48] McDonald E. M. (2020). TUNEL apoptotic cell detection in stony coral tissue loss disease. SCTLD: Evaluation of Potential And Improvements.

[ref49] McManus L. C. Forrest D. L. Tekwa E. W. Schindler D. E. Colton M. A. Webster M. M. . (2021). Evolution and connectivity influence the persistence and recovery of coral reefs under climate change in the Caribbean, Southwest Pacific, and coral triangle. Glob. Chang. Biol. 27, 4307–4321. 34106494 10.1111/gcb.15725 PMC8453988

[ref50] Million W. C. Voolstra C. R. Perna G. Puntin G. Rowe K. Ziegler M. (2025). Resolving Symbiodiniaceae diversity across coral microhabitats and reef niches. Environ. Microbiol. 27:e70065. 40038092 10.1111/1462-2920.70065 PMC11879917

[ref51] Mote S. Gupta V. De K. Hussain A. More K. Nanajkar M. . (2021). Differential Symbiodiniaceae association with coral and coral-eroding sponge in a bleaching impacted marginal coral reef environment. Front. Mar. Sci. 8:666825.

[ref52] Mouillot D. Villéger S. Parravicini V. Kulbicki M. Arias-González J. E. Bender M. . (2014). Functional over-redundancy and high functional vulnerability in global fish faunas on tropical reefs. Proc. Natl. Acad. Sci. 111, 13757–13762. 25225388 10.1073/pnas.1317625111 PMC4183327

[ref53] Nitschke M. R. Rosset S. L. Oakley C. A. Gardner S. G. Camp E. F. Suggett D. J. . (2022). The diversity and ecology of Symbiodiniaceae: a traits-based review. Adv. Mar. Biol. 92, 55–127. 36208879 10.1016/bs.amb.2022.07.001

[ref54] Noonan K. R. Childress M. J. (2020). Association of butterflyfishes and stony coral tissue loss disease in the Florida keys. Coral Reefs 39, 1581–1590.

[ref55] Parker G. M. (1984). Dispersal of zooxanthellae on coral reefs by predators on cnidarians. Biol. Bull. 167, 159–167.

[ref56] Pratchett M. S. Hoey A. S. Feary D. A. Bauman A. G. Burt J. A. Riegl B. M. (2013). Functional composition of Chaetodon butterflyfishes at a peripheral and extreme coral reef location, the Persian Gulf. Mar. Pollut. Bull. 72, 333–341. 23140852 10.1016/j.marpolbul.2012.10.014

[ref57] R Core Team (2022). R: A language and environment for statistical computing. Vienna: R Foundation for Statistical Computing https://www.R-project.org.

[ref58] Rotjan R. D. Lewis S. M. (2008). Impact of coral predators on tropical reefs. Mar. Ecol. Prog. Ser. 367, 73–91.

[ref59] Santos S. R. Shearer T. L. Hannes A. R. Coffroth M. A. (2004). Fine-scale diversity and specificity in the most prevalent lineage of symbiotic dinoflagellates (Symbiodinium, Dinophyceae) of the Caribbean. Mol. Ecol. 13, 459–469. 14717900 10.1046/j.1365-294x.2003.02058.x

[ref60] Scharfenstein H. J. Chan W. Y. Buerger P. Humphrey C. van Oppen M. J. (2022). Evidence for de novo acquisition of microalgal symbionts by bleached adult corals. ISME J. 16, 1676–1679. 35132118 10.1038/s41396-022-01203-0 PMC9122906

[ref61] Schiettekatte N. M. Casey J. M. Brandl S. J. Mercière A. Degregori S. Burkepile D. . (2023). The role of fish feces for nutrient cycling on coral reefs. Oikos 2023:e09914.

[ref62] Shirur K. P. Ramsby B. D. Iglesias-Prieto R. Goulet T. L. (2014). Biochemical composition of Caribbean gorgonians: implications for gorgonian—Symbiodinium symbiosis and ecology. J. Exp. Mar. Biol. Ecol. 461, 275–285.

[ref63] Silverstein R. N. Correa A. Baker A. C. (2012). Specificity is rarely absolute in coral–algal symbiosis: implications for coral response to climate change. Proc. R. Soc. B Biol. Sci. 279, 2609–2618. 10.1098/rspb.2012.0055 PMC3350700 22367985

[ref64] Speare K. E. Adam T. C. Winslow E. M. Lenihan H. S. Burkepile D. E. (2022). Size-dependent mortality of corals during marine heatwave erodes recovery capacity of a coral reef. Glob. Chang. Biol. 28, 1342–1358. 34908214 10.1111/gcb.16000

[ref65] Swaminathan S. D. Lafferty K. D. Knight N. S. Altieri A. H. (2024). Stony coral tissue loss disease indirectly alters reef communities. *Science*. Advances 10:eadk6808. 10.1126/sciadv.adk6808 PMC11068009 38701216

[ref66] Terraneo T. I. Fusi M. Hume B. C. Arrigoni R. Voolstra C. R. Benzoni F. . (2019). Environmental latitudinal gradients and host-specificity shape Symbiodiniaceae distribution in Red Sea Porites corals. J. Biogeogr. 46, 2323–2335.

[ref67] Teschima M. M. Garrido A. Paris A. Nunes F. L. Zilberberg C. (2019). Biogeography of the endosymbiotic dinoflagellates (Symbiodiniaceae) community associated with the brooding coral *Favia gravida* in the Atlantic Ocean. PLoS One 14:e0213519. 30849101 10.1371/journal.pone.0213519 PMC6407780

[ref68] Titus B. M. Daly M. Exton D. A. (2019). Temporal patterns of coral reef benthic communities in the Caribbean: the role of marine reserves. Mar. Ecol. Prog. Ser. 627, 73–85.

[ref69] Titus K. O’Connell L. Matthee K. Childress M. (2022). The influence of foureye butterflyfish (*Chaetodon capistratus*) and symbiodiniaceae on the transmission of stony coral tissue loss disease. Front. Mar. Sci. 9:800423.

[ref70] Tremblay P. Gori A. Maguer J. F. Hoogenboom M. Ferrier-Pagès C. (2016). Heterotrophy promotes the re-establishment of photosynthate translocation in a symbiotic coral after heat stress. Sci. Rep. 6:38112. 27917888 10.1038/srep38112 PMC5137022

[ref71] Vlam O. (2024). Functional role of herbivorous fish on the reef of Bonaire (Master’s thesis).: Utrecht University Student Theses Repository.

[ref72] Wainwright P. C. Price S. A. (2018). “Innovation and diversity of the feeding mechanism in parrotfishes” in Biology of parrotfishes (Boca Raton, Florida, USA: CRC Press), 26–41.

[ref73] Wickham H. Chang W. Wickham M. H. (2016). Package ‘ggplot2’. *Create elegant data visualisations using the grammar of graphics*. Version 2, 1–189.

[ref74] Williamson O. M. Allen C. E. Williams D. E. Johnson M. W. Miller M. W. Baker A. C. (2021). Neighboring colonies influence uptake of thermotolerant endosymbionts in threatened Caribbean coral recruits. Coral Reefs 40, 867–879.

[ref75] Winkler N. S. Pandolfi J. M. Sampayo E. M. (2015). Symbiodinium identity alters the temperature-dependent settlement behaviour of *Acropora millepora* coral larvae before the onset of symbiosis. Proc. R. Soc. B Biol. Sci. 282:20142260. 10.1098/rspb.2014.2260 PMC4308998 25589607

