# Supplementary material for: Caribbean fish feces are an environmental hotspot of viable Symbiodiniaceae
Source: Front Microbiol. 2026 Feb 12;16:1715855. doi: 10.3389/fmicb.2025.1715855 (PMC12937136; doi:10.3389/fmicb.2025.1715855)
Supplement: Supplementary file 1 [file Data_Sheet_1.pdf]

# Supplementary Material

Data and code are available at https://github.com/karatitus/Caribbean-Fish-Feces-2025

**Figure S1.** A) Benthic composition, fish behavior, and Symbiodiniaceae diversity and density were assessed at four U.S. Virgin Islands reef sites (red dots): Buck Island and Cane Bay (St. Croix, SC, dots 1 and 2) and Coral Bay and South Haulover (St. John, SJ, dots 3 and 4) in 2021 and 2022. At the time of sampling, each site constituted a stony coral tissue loss disease (SCTLD) endemic zone (Brandt et al. 2021). B) Summary of fish fecal and coral samples collected, processed, and statistically analyzed to quantify Symbiodiniaceae assemblages from U.S. Virgin Island reefs. BF – butterflyfish, PF – parrotfish, SF – surgeonfish, ACBA – *Acanthurus bahianus,* ACCE – *Acropora cervicornis,* ACCO – *Acanthurus coeruleus,* AGAG – *Agaricia agaricites,* CHCA – *Chaetodon capistratus,* CHST – *Chaetodon striatus,* CONA – *Colpophyllia natans,* DILA – *Diploria labyrinthiformis,* MOCA – *Montastraea cavernosa,* ORAN – *Orbicella annularis,* POAS – *Porites astreoides,* SPAU – *Sparisoma aurofrenatum,* SPVI – *Sparisoma viride.*

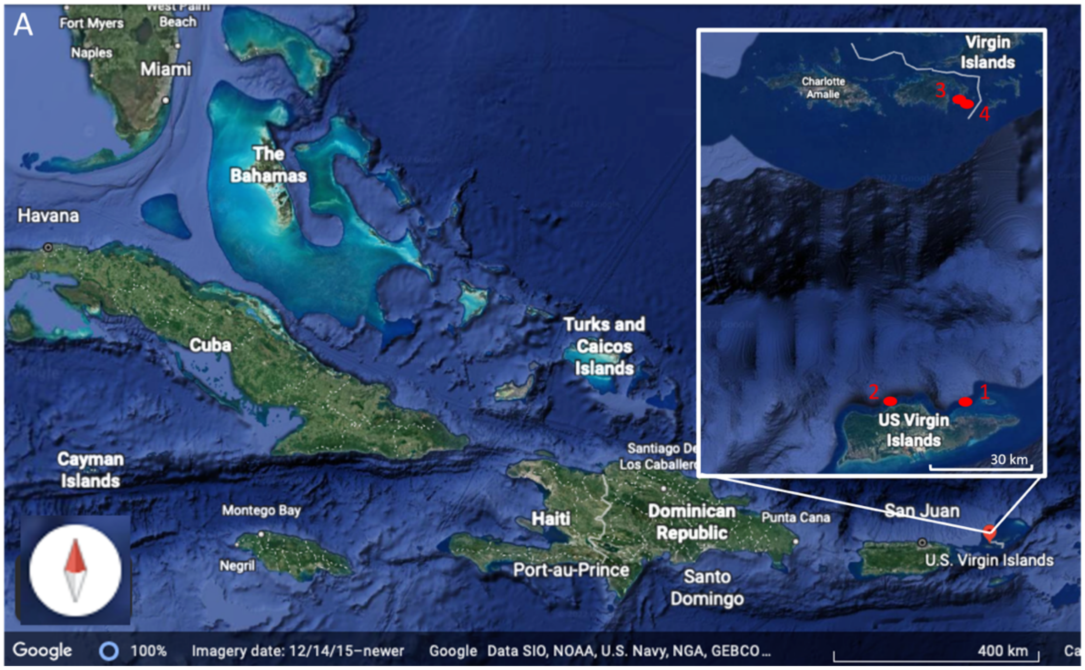


| B<br>**Sample type** | | **Coral Bay (SJ)** | **South Haulover (SJ)** | **Buck Island (SC)** | **Cane Bay (SC)** |
|---|---|---|---|---|---|
| **Feces from Fish spp.▼** | **Codes** | **Symbiodiniaceae cell density** | | | |
| *Chaetodon capistratus* | BF, CHCA | 2 | 3 | 1 | 0 |
| *Chaetodon striatus* | BF, CHST | 0 | 0 | 1 | 4 |
| *Sparisoma aurofrenatum* | PF, SPAU | 2 | 4 | 4 | 3 |
| *Sparisoma viride* | PF, SPVI | 3 | 3 | 0 | 0 |
| *Acanthurus bahianus* | SF, ACBA | 3 | 1 | 0 | 0 |
| *Acanthurus coeruleus* | SF, ACCO | 5 | 0 | 0 | 4 |
| **Feces from Fish spp.▼** | | **Symbiodiniaceae diversity** | | | |
| *Chaetodon capistratus* | BF, CHCA | 2 | 3 | 1 | 4 |
| *Chaetodon striatus* | BF, CHST | 0 | 0 | 1 | 4 |

| | | | | | |
|---|---|---|---|---|---|
| *Sparisoma aurofrenatum* | PF, SPAU | 2 | 3 | 2 | 2 |
| *Sparisoma viride* | PF, SPVI | 2 | 3 | 1 | 0 |
| *Acanthurus bahianus* | SF, ACBA | 3 | 1 | 0 | 4 |
| *Acanthurus coeruleus* | SF, ACCO | 5 | 0 | 0 | 3 |
| **Coral Tissue ▼** | | | | | |
| *Acropora cervicornis* | ACCE | 0 | 0 | 0 | 5 |
| *Agaricia agaricites* | AGAG | 8 | 9 | 12 | 15 |
| *Colpophyllia natans* | CONA | 13 | 12 | 15 | 12 |
| *Diploria labyrinthiformis* | DILA | 8 | 6 | 5 | 6 |
| *Montastraea cavernosa* | MOCA | 10 | 8 | 6 | 15 |
| *Orbicella annularis* | ORAN | 8 | 6 | 8 | 14 |
| *Porites astreoides* | POAS | 9 | 11 | 11 | 12 |

**Table S1.** Symbiodiniaceae internal transcribed spacer-2 (ITS-2) type profiles recovered from Caribbean coral tissues and fish feces in this study are organized based on the dominant defining intragenomic variants (DIVs), following the framework described by Hume et al. (2019). A slash (/) between DIVs (e.g., A3/A4) indicates co-dominance, where both variants are present in similar relative abundances. In contrast, a hyphen (-) denotes a dominant DIV followed by one or more subdominant, consistently co-occurring intragenomic variants (e.g., A3-A3ac-A3t), which are a part of the same symbiont genotype but occur at lower relative abundance.

| **Dominant DIV** | **Symbiodiniaceae ITS-2 Type Profile** |
|---|---|
| A1dh | A1dh |
| A2l | A2l |
| A2w | A2w |
| A3 | A3 |
| | A3-A3at-A3bi |
| | A3-A3m |
| | A3-A3t-A3ad |
| | A3-A3t-A3ad-A3ac |
| A3/A4 | A3/A4 |
| A3/A4/A4a | A3/A4/A4a |
| A4 | A4 |
| | A4-A4a-A4bf |
| | A4-A4bs-A4bw-A4bx |
| | A4-A4de |
| | A4-A4t |
| A4/A4a | A4/A4a |
| | A4/A4a-A4dz |
| | A4a/A4-A4.3 |
| A4/A4a/A4bz | A4/A4a/A4bz |
| A4/A4a/A4cb | A4/A4a/A4cb |
| | A4a/A4/A4cb-A4dz |
| A4/A4ea | A4/A4ea-A4a |
| A4cz | A4cz |
| A4df | A4df |
| | A4df-A4cz-A4-A4eb-A4dg |
| A4z | A4z |
| A13 | A13 |
| B1 | B1 |

| | |
|---|---|
| | B1-B14k-B1fe |
| | B1-B1a1-B1ak-B1aj-B1aa-B1gj |
| | B1-B1ca |
| | B1-B1cg |
| | B1-B1cx-B1fk-B1gk |
| | B1-B1cx-B1fm-B10-B1fk |
| | B1-B1by |
| | B1-B1do-B10 |
| | B1-B1do-B1fk-B1cx |
| | B1-B1ed |
| | B1-B1fi-B1bh-B1fj |
| | B1-B1g |
| | B1-B1gl-B1ah-B1aa |
| | B1-B1gm-B1gn-B14b |
| | B1-B1x |
| | B1-B1x-B1ag |
| | B1-B1x-B1t-B1ae-B1af-B14d-B1ag-B1az-B14b |
| B1/B5 | B1/B5 |
| | B5/B1-B1do |
| B1/B19 | B1/B19 |
| | B1/B19-B5af |
| B1/B1do | B1/B1do |
| B2/B1 | B2/B1 |
| B2d | B2d |
| B5 | B5 |
| B7 | B7 |
| | B7-B7d |
| B8a | B8a |
| | B8a-B8g |
| B18e | B18e |
| B18e/B7/B1 | B18e/B7/B1 |
| B19 | B19 |
| | B19-B19ba |
| | B19-B5af-B19e-B19az-B19f-B5ag |
| | B19-B5af-B19e-B19g-B19az-B19d-B5ag-B5du |
| | B19-B5af-B19e-B5dt-B19d-B19h-B5ar-B5ag |
| | B19-B5af-B19e-B19c-B19d-B19f-B5ag-B40a |
| B19/B5af | B19/B5af-B19e-B19d-B5ag-B19f-B19c-B40a-B19h |
| B19bb | B19bb |
| B19bc | B19bc |
| C1 | C1-C1b-C1c-C42.2-C1bh-C1br-C3-C1cb |
| | C1-C1c-C1b-C3-C42.2-C1bh |
| | C1-C1c-C1b-C3-C42.2-C1bh-C1br |
| | C1-C21-C3-C1c-C1b-C42.2 |
| | C1-C1c-C3 |
| C1/C1c | C1/C1c |
| | C1/C1c-C3-C1al |
| | C1/C1c-C1al-C1b-C42.2 |
| C1/C1c/C3 | C1/C1c/C3-C1b-C72k |
| C1/C3 | C1/C3-C1c-C1b-C42.2-C1br |
| | C1/C3-C42.2-C1c |
| C1/C7 | C1/C7 |
| C1/C42.2 | C1/C42.2-C1c-C1b-C72k |

| | |
|---|---|
| C1/C42.2/C1b/C3 | C1/C42.2/C1b/C3 |
| C1/C42.2/C3/C1c | C1/C42.2/C3/C1c-C1b |
| C3 | C3-C3an-C21-C3b-C3fc-C3s |
| | C3-C3de-C21-C3bb-C3an-C3b-C21ae-C3s |
| | C3-C3de-C3bb-C21ae-C3an-C35-C21-C3dk-C3ji |
| | C3-C3fc-C21-C3b-C3an-C3fd-C35-C3bb |
| | C3-C21-C3an-C3b |
| | C3-C40g-C3fc-C3b-C3an-C21-C3fd-C3s |
| C3/C3b | C3/C3b |
| | C3b/C3-C21-C3s-C3bb-C3abb-C3e-C3ag |
| C3/C3fe | C3/C3fe-C3-C3fg |
| C3/C3u | C3/C3u |
| C3b | C3b-C3-C21-C3s-C3bb-C3abb-C3ag |
| | C3b-C3-C21-C3ag-C3bb-C3s |
| C3b/C3/C3abc | C3b/C3/C3abc-C21-C3s-C3ag |
| C3b/C3fe | C3b/C3fe-C3-C3fg |
| C3fe | C3fe-C3-C3fg-C3b-C3ag-C21-C31r |
| C3u | C3u |
| C42.2/C45a | C42.2/C45a-C42fb-C44i |
| C44 | C44 |
| C54b/C54a | C54b/C54a-C3-C54d-C54c |
| C66 | C66 |
| C7 | C7-C12c-C7d-C12b |
| C7/C12c/C7d | C7/C12c/C7d-C12b-C12e-C7c |
| | C7/C12c/C7d-C12b-C12e-C7c-C7b |
| C7/C3 | C7/C3-C12c-C7d |
| C12c/C7 | C12c/C7-C7d-C12b-C12e-C7h |
| C12c/C7/C7d | C12c/C7/C7d-C12b-C12e-C7h-C12f |
| C21/C1 | C21/C1 |
| C80 | C80 |
| C80b | C80b |
| C91 | C91 |
| D1 | D1-D4-D4c-D1c-D1k-D17d |
| | D1-D4-D4c-D1c-D1k-D2 |
| | D1-D4-D4c-D2-D4f-D3b |
| | D1-D4-D4c-D6-D1c |
| D1/D4/D2 | D1/D4/D2-D4c-D1c |
| D1/D4/D4c | D1/D4/D4c |
| F5.1 | F5.1 |
| F5bf | F5bf |

**Figure S2.** A) The percent cover of macroalgae, gorgonians, sponges, stony corals, rock/sand, and other substrates were averaged at each of two sites in St. Croix (Buck Island and Cane Bay), and at two sites in St. John (Coral Bay and South Haulover). Disease prevalence data for stony corals are based on video transect data. B) Results of pairwise PERMANOVA test on the benthic compositions between sites. Significant *p*-values ($< 0.05$) are bolded. Overall PERMANOVA test results: df = 3.20, F = 15.992, $p = 0.001$.

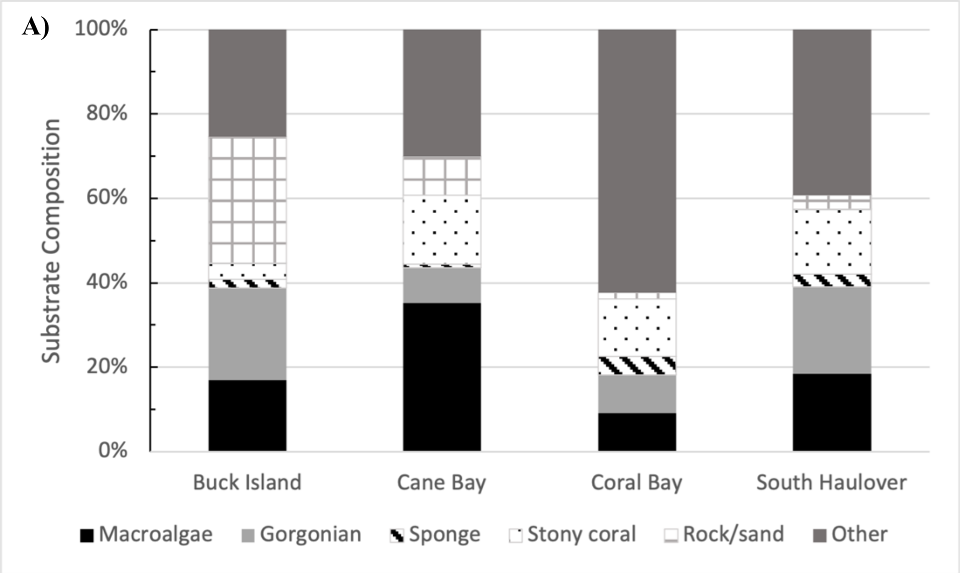


| B) Pairwise Comparisons | Sum of Squares | F Model | $R^2$ | Adjusted *p*-value |
|---|---|---|---|---|
| Buck Island vs Cane Bay | 0.3115 | 18.2542 | 0.6461 | **0.018** |
| Buck Island vs South Haulover | 0.2643 | 16.4195 | 0.6215 | **0.006** |
| Buck Island vs Coral Bay | 0.3267 | 22.5297 | 0.6926 | **0.018** |
| Cane Bay vs South Haulover | 0.1358 | 11.0741 | 0.5255 | **0.030** |
| Cane Bay vs Coral Bay | 0.1594 | 14.9408 | 0.5991 | **0.006** |
| South Haulover vs Coral Bay | 0.0864 | 8.9030 | 0.4710 | **0.012** |

**Table S2.** Bites (totals and percent) and defecations (totals) observed by the six Caribbean fish species across reef substrate types during fish follows. Numbers in parentheses following fish species codes indicate the total number of fish followed. Coral tissue group categories are organized by healthy tissue on healthy corals (HH), healthy tissue on diseased corals (HD), and diseased tissue on diseased corals (DD). Caribbean corals – CCor, Caribbean herbivore/detritivore – CH, and Caribbean facultative corallivore – CF. ACBA (CH) – *Acanthurus bahianus,* ACCO (CH) – *Acanthurus coeruleus,* AGAG (CCor) – *Agaricia agaricites,* CHCA (CF) – *Chaetodon capistratus,* CHST (CF) – *Chaetodon striatus,* CONA (CCor) – *Colpophyllia natans,* DILA (CCor) – *Diploria labyrinthiformis,* MASP (CCor) – *Madracis* sp., MISP (CCor) – *Millepora* sp., MOCA (CCor) – *Montastraea cavernosa,* ORAN (CCor) – *Orbicella annularis,* ORFA (CCor) – *Orbicella faveolata,* ORFR (CCor) – *Orbicella franksi,* POAS (CCor) – *Porites astreoides,* POPO (CCor) – *Porites porites,* SISI (CCor) – *Siderastrea siderea,* SPAU (CF) – *Sparisoma aurofrenatum,* and SPVI (CF) – *Sparisoma viride.*

| Caribbean Fish Species ▶ | | CHCA (23) | CHST (11) | SPAU (22) | SPVI (20) | ACBA (19) | ACCO (18) |
|---|---|---|---|---|---|---|---|
| | | **Facultative Corallivore** | | | | **Herbivore/Detritivore** | |
| **Caribbean Coral Species ▼** | | **Total # of Bites** | | | | | |
| AGAG | HH | 5 | 0 | 0 | 0 | 0 | 0 |
| CONA | HH | 1 | 0 | 0 | 0 | 0 | 0 |
| | HD | 3 | 0 | 0 | 0 | 0 | 0 |
| | DD | 3 | 0 | 0 | 0 | 0 | 0 |
| DILA | HD | 2 | 0 | 0 | 0 | 0 | 0 |
| MASP | HH | 0 | 0 | 0 | 1 | 0 | 0 |
| MISP | HH | 0 | 0 | 19 | 0 | 0 | 1 |
| MOCA | HH | 1 | 0 | 0 | 0 | 0 | 1 |
| ORAN | HH | 77 | 0 | 0 | 5 | 0 | 5 |
| | HD | 19 | 0 | 0 | 3 | 1 | 0 |
| | DD | 40 | 0 | 0 | 0 | 0 | 0 |
| ORFA | HH | 0 | 0 | 0 | 1 | 0 | 0 |
| ORFR | HH | 14 | 0 | 0 | 8 | 1 | 0 |
| | HD | 0 | 0 | 4 | 0 | 0 | 0 |
| | DD | 0 | 0 | 0 | 0 | 0 | 0 |
| POAS | HH | 16 | 0 | 0 | 0 | 0 | 0 |
| POPO | HH | 0 | 0 | 0 | 0 | 0 | 1 |
| SISI | HH | 8 | 0 | 0 | 0 | 0 | 0 |
| Unknown Stony Coral | -- | 129 | 2 | 2 | 3 | 1 | 1 |
| Soft Coral | -- | 71 | 4 | 52 | 33 | 39 | 9 |
| **Caribbean Other Substrates ▼** | | | | | | | |
| Algae | -- | 45 | 38 | 290 | 222 | 455 | 310 |
| Rock/sand | -- | 50 | 18 | 470 | 523 | 857 | 405 |
| Sponge | -- | 0 | 0 | 1 | 0 | 17 | 3 |
| Other | -- | 44 | 0 | 0 | 6 | 8 | 13 |
| **Total Bites** | | 528 | 62 | 838 | 805 | 1379 | 749 |
| **Average Bites per Follow** | | 23 | 6 | 38 | 40 | 73 | 42 |
| | | **% Bites from Substrate Types** | | | | | |
| % Bites Algae | -- | 8.52% | 61.29% | 34.61% | 27.58% | 32.99% | 41.39% |
| % Bites Rock/Sand | -- | 9.47% | 29.03% | 56.09% | 64.97% | 62.15% | 54.07% |
| % Bites Soft Coral | -- | 13.45% | 6.45% | 6.21% | 4.10% | 2.83% | 1.20% |
| % Bites Sponge | | 0.00% | 0.00% | 0.12% | 0.00% | 1.23% | 0.40% |
| % Bites Stony Coral | -- | 60.23% | 3.23% | 2.98% | 2.61% | 0.22% | 1.20% |
| % Bites Other | -- | 8.33% | 0.0% | 0.0% | 0.75% | 0.58% | 1.74% |
| | | **Total Defecations** | | | | | |
| Defecations | -- | 5 | 0 | 30 | 17 | 5 | 7 |

**Table S3.** Percent of bites taken across different substrate types from the fish follow surveys conducted on nine Pacific fish species from Grupstra et al. (2021). ACSP – *Acropora* sp., AMSC – *Amanses scopas,* CHCI – *Chaetodon citrinellus,* CHLU – *Chaetodon lunulatus,* CHOR – *Chaetodon ornatissimus,* CHPE – *Chaetodon pelewensis,* CHRE – *Chaetodon reticulatus,* CHSP – *Chlolurus spilurus,* CTFL – *Ctenochaetus flavicauda,* CTST – *Ctenochaetus striatus,* MOSP – *Montipora* sp., POSP – *Pocillopora* sp.

| **Pacific Fish Species ▶** | **AMSC** | **CHLU** | **CHOR** | **CHRE** | **CHCI** | **CHPE** | **CHSP** | **CTFL** | **CTST** |
|---|---|---|---|---|---|---|---|---|---|
| | Obligate corallivores | | | | Facultative corallivores | | | Herbivores | |
| **Substrate Types ▼** | **% Bites from Substrate Types** | | | | | | | | |
| ACSP | 3.2% | 0.8% | 0.6% | 2.2% | 1.5% | 2.0% | 0.0% | 0.0% | 0.0% |
| MOSP | 6.7% | 20.0% | 15.4% | 10.5% | 9.1% | 2.6% | 1.6% | 0.4% | 0.0% |
| POSP | 87.8% | 9.7% | 50.7% | 67.3% | 8.8% | 82.8% | 2.0% | 0.2% | 0.0% |
| Other Stony Coral | 0.4% | 64.5% | 31.4% | 17.6% | 6.9% | 6.5% | 0.7% | 0.0% | 0.1% |
| Macroalgae | 0.4% | 1.0% | 0.1% | 0.0% | 31.2% | 0.9% | 7.8% | 2.6% | 8.3% |
| Turf on Rock | 1.2% | 3.4% | 0.9% | 1.6% | 41.6% | 3.8% | 86.3% | 96.5% | 91.5% |
| Other | 0.0% | 0.3% | 0.4% | 0.3% | 0.6% | 0.9% | 1.2% | 0.0% | 0.0% |

**Table S4.** Results of Dunn's test on the total bites taken by Caribbean fish species (Table 1). Significant *p*-values (< 0.05) are bolded. Foraging group categories are organized by herbivore/detritivore (H) and facultative corallivore (F). ACBA – *Acanthurus bahianus,* ACCO – *Acanthurus coeruleus,* CHCA – *Chaetodon capistratus,* CHST – *Chaetodon striatus,* SPAU – *Sparisoma aurofrenatum,* and SPVI – *Sparisoma viride.* Overall Kruskal-Wallis test results: $X^2 = 14.267$, df = 5, $p = 0.014$.

| **Species Comparison** | **Foraging Group** | **Z statistic** | **Adjusted *p*-value** |
|---|---|---|---|
| ACBA - ACCO | H-H | 0.4331 | 0.4987 |
| ACBA-CHCA | H-F | 0.5626 | 0.5379 |
| ACCO-CHCA | H-F | 0.1016 | 0.4924 |
| ACBA-CHST | H-F | 3.2251 | **0.0031** |
| ACCO-CHST | H-F | 2.8205 | **0.0072** |
| CHCA-CHST | F-F | 2.8573 | **0.0080** |
| ACBA-SPAU | H-F | 0.0662 | 0.4736 |
| ACCO-SPAU | H-F | -0.3829 | 0.4785 |
| CHCA-SPAU | F-F | -0.5153 | 0.5053 |
| CHST-SPAU | F-F | -3.2527 | **0.0043** |
| ACBA-SPVI | H-F | -0.1822 | 0.4935 |
| ACCO-SPVI | H-F | -0.6182 | 0.5748 |
| CHCA-SPVI | F-F | -0.7614 | 0.5580 |
| CHST-SPVI | F-F | -3.4106 | **0.0049** |
| SPAU-SPVI | F-F | -0.2561 | 0.4987 |

**Table S5.** Results of Dunn's test on the total defecations taken by Caribbean fish species during follows (Table 1). Significant *p*-values (< 0.05) are bolded. Foraging group categories are organized by herbivore/detritivore (H) and facultative corallivore (F). ACBA – *Acanthurus bahianus,* ACCO – *Acanthurus coeruleus,* CHCA – *Chaetodon capistratus,* CHST – *Chaetodon striatus,* SPAU – *Sparisoma aurofrenatum,* and SPVI – *Sparisoma viride.* Overall Kruskall-Wallis test results across fish species: $X^2 = 21.6992$, df = 5, $p < 0.001$.

| Species Comparison | Foraging Group | Z statistic | Adjusted *p*-value |
|---|---|---|---|
| ACBA-ACCO | H-H | 0.0897 | 0.4642 |
| ACBA-CHCA | H-F | -1.0973 | 0.1572 |
| ACCO-CHCA | H-F | 1.1748 | 0.1637 |
| ACBA-CHST | H-F | -1.4025 | 0.1507 |
| ACCO-CHST | H-F | -1.4656 | 0.1530 |
| CHCA-CHST | F-F | -0.5215 | 0.3225 |
| ACBA-SPAU | H-F | -2.5567 | **0.0264** |
| ACCO-SPAU | H-F | -2.4266 | **0.0229** |
| CHCA-SPAU | F-F | -3.8258 | **0.0010** |
| CHST-SPAU | F-F | -3.6073 | **0.0012** |
| ACBA-SPVI | H-F | -1.2454 | 0.1598 |
| ACCO-SPVI | H-F | -1.1371 | 0.1597 |
| CHCA-SPVI | F-F | -2.4536 | **0.0195** |
| CHST-SPVI | F-F | -2.4784 | **0.0247** |
| SPAU-SPVI | F-F | 1.3304 | 0.1612 |

**Table S6.** Symbiodiniaceae live and dead cell density ranges and means in feces of the six species of Caribbean fishes (Figure 1). Foraging group (FG) categories are organized by herbivore/detritivore (H) and facultative corallivore (F). ACBA – *Acanthurus bahianus,* ACCO – *Acanthurus coeruleus,* CHCA – *Chaetodon capistratus,* CHST – *Chaetodon striatus,* SPAU – *Sparisoma aurofrenatum,* and SPVI – *Sparisoma viride.*

| | | **Dead Cell Densities** | | **Live Cell Densities** | |
|---|---|---|---|---|---|
| **Species** | **FG** | **Density Range** | **Mean** | **Density Range** | **Mean** |
| CHCA | F | 32,740 – 21,895,815 | 4,315,634 ± 8,681,683 | 2,476,346 – 26,425,500 | 8,228,097 ± 9,324,969 |
| CHST | F | 38,250 - 4,041,750 | 879,225 ± 1,769,094 | 126,000 – 1,080,000 | 706,125 ± 372,988 |
| SPAU | F | 35,625 - 8,163,000 | 1,090,359 ± 2,255,945 | 114,000 – 19,755,000 | 5,622,494 ± 6,669,345 |
| SPVI | F | 143,379 - 729,522 | 489,681 ± 243,290 | 2,023,241 – 11,611,911 | 7,067,178 ± 4,301,970 |
| ACBA | H | 30,010 – 323,574 | 167,968 ± 147,575 | 439,235 – 2,706,972 | 1,231,626 ± 1,278,858 |
| ACCO | H | 76,781 – 792,714 | 591,175 ± 533,125 | 1,143,188 – 5,463,000 | 2,927,157 ± 1,228,263 |

**Table S7.** Results of Dunn's tests comparing Symbiodiniaceae densities in the feces of six Caribbean fish species (Figure 1). Benjamini-Hochberg procedure was applied to control for false positives, no *p*-values were significant (<0.05). Foraging group categories are organized by herbivore/detriticore (H) and facultative corallivore (F). ACBA – *Acanthurus bahianus,* ACCO – *Acanthurus coeruleus,* CHCA – *Chaetodon capistratus,* CHST – *Chaetodon striatus,* SPAU – *Sparisoma aurofrenatum,* and SPVI – *Sparisoma viride.* Overall Kruskall-Wallis test results for live Symbiodiniaceae cells across fish species: $X^2 = 16.9866$, df = 5, $p = 0.0045$. Overall Kruskall-Wallis test results for dead Symbiodiniaceae cells across fish species: $X^2 = 5.1765$, df = 5, $p = 0.3947$.

| | | Dead Symbiodiniaceae cells | | Live Symbiodiniaceae cells | |
|---|---|---|---|---|---|
| **Species Comparison** | **Foraging Group** | **Z statistic** | **Adjusted *p*-value** | **Z statistic** | **Adjusted *p*-value** |
| ACBA-ACCO | H-H | -1.572176 | 0.4347 | -1.516524 | 0.1213 |
| ACBA-CHCA | H-F | -1.396999 | 0.174 | -2.302097 | **0.0267** |
| ACCO-CHCA | H-F | 0.114392 | 0.5244 | -1.170319 | 0.1512 |
| ACBA-CHST | H-F | -0.335301 | 0.6145 | 0.426747 | 0.3863 |
| ACCO-CHST | H-F | 1.440096 | 0.281 | 2.371337 | **0.0266** |
| CHCA-CHST | F-F | 1.226954 | 0.2061 | 3.202947 | **0.0051** |
| ACBA-SPAU | H-F | -1.562666 | 0.2953 | -1.594997 | 0.1186 |
| ACCO-SPAU | H-F | 0.089396 | 0.4976 | -0.042069 | 0.4832 |
| CHCA-SPAU | F-F | -0.041739 | 0.4834 | 1.196523 | 0.1578 |
| CHST-SPAU | F-F | -1.434981 | 0.2269 | -2.519712 | 0.0294 |
| ACBA-SPVI | H-F | -1.574083 | 0.866 | -2.420153 | 0.0291 |
| ACCO-SPVI | H-F | -0.123191 | 0.5637 | -1.328708 | 0.138 |
| CHCA-SPVI | F-F | -0.216883 | 0.6212 | -0.144588 | 0.4741 |
| CHST-SPVI | F-F | -1.433744 | 0.1896 | -3.340807 | **0.0063** |
| SPAU-SPVI | F-F | -0.208695 | 0.5691 | -1.36348 | 0.1439 |

**Table S8.** Results of Kruskall-Wallis test on the densities of live and dead Symbiodiniaceae cells in the feces of Caribbean fish based on fish size, fish weight, fish phase, and reef site.

| Variable | Live Cells | Dead Cells |
|---|---|---|
| Fish Size (cm) | $X^2 = 28.81$, df = 31, $p = 0.579$ | $X^2 = 30.42$, df = 29, $p = 0.393$ |
| Fish weight (g) | $X^2 = 47$, df = 47, $p = 0.473$ | $X^2 = 1.04$, df = 47, $p = 0.309$ |
| Fish phase | $X^2 = 1.02$, df = 1, $p = 0.314$ | $X^2 = 0.01$, df = 1, $p = 0.919$ |
| Reef Site | $X^2 = 1.10$, df = 3, $p = 0.777$ | $X^2 = 1.22$, df = 3, $p = 0.747$ |

**Table S9.** Results of pairwise Dunn tests on the densities of Caribbean Symbiodiniaceae and Pacific Symbiodiniaceae in fish feces between foraging groups (Figure 1). Benjamini-Hochberg procedure was used to control for false positives. Significant *p*-values (<0.05) are bolded. Foraging group categories are organized by Caribbean herbivore/detritivore (CH), Caribbean facultative corallivore (CF), Pacific herbivore/detritivore (PH), Pacific facultative corallivore (PF), Pacific obligate corallivore (PO), and Pacific environmental sample (PE). Overall Kruskall-Wallis test results for live Symbiodiniaceae cells across region and foraging groups: $X^2 = 88.5734$, df = 5, $p < 0.001$. Overall Kruskall-Wallis test results for dead Symbiodiniaceae cells across region and foraging groups: $X^2 = 84.9118$, df = 5, $p < 0.001$.

| | Dead Symbiodiniaceae cells | | Live Symbiodiniaceae cells | |
|---|---|---|---|---|
| **Foraging Group** | **Z statistic** | **Adjusted *p*-value** | **Z statistic** | **Adjusted *p*-value** |
| CF-PO | **-3.2553** | **0.0012** | -1.0832 | 0.1608 |
| CF-PF | **-2.9900** | **0.0023** | **2.3491** | **0.0141** |
| CF-PH | 0.9233 | 0.2224 | **5.0124** | **<0.001** |
| CH-PO | **-2.8388** | **0.0034** | -1.5663 | 0.0799 |
| CH-PF | **-2.7469** | **0.0041** | 1.1503 | 0.1563 |
| CH-PH | 0.4069 | 0.3665 | **3.5077** | **<0.001** |
| CF-PE | **4.8799** | **<0.001** | **6.4159** | **<0.001** |
| CH-PE | **3.4117** | **<0.001** | **4.2968** | **<0.001** |

**Figure S3.** A) Results of pairwise PERMANOVA of Symbiodiniaceae assemblages varied significantly between Caribbean coral species and fish feces based on the Symbiodiniaceae ITS-2. Foraging group categories are organized by coral (Cor), herbivore/detritivore (H), and facultative corallivores (F). B) Shannon diversity of Symbiodiniaceae assemblages recovered from fish feces and coral tissue samples. Fish fecal communities generally exhibit higher diversity relative to coral hosts. ACBA – *Acanthurus bahianus,* ACCE – *Acropora cervicornis,* ACCO – *Acanthurus coeruleus,* AGAG – *Agaricia agaricites,* CHCA – *Chaetodon capistratus,* CHST – *Chaetodon striatus,* CONA – *Colpophyllia natans,* DILA – *Diploria labyrinthiformis,* MOCA – *Montastraea cavernosa,* ORAN – *Orbicella annularis,* POAS – *Porites astreoides,* SPAU – *Sparisoma aurofrenatum,* SPVI – *Sparisoma viride*. Overall PERMANOVA test results: df = 7, F = 28.298, $p < 0.001$.

| A. Species | Sample Types | Foraging Group | Sum of Squares | $R^2$ | F Statistic | Adjusted *p*-value |
|---|---|---|---|---|---|---|
| AGAG-ACBA | Coral-Feces | Cor-H | 0.7804 | 0.1976 | 2.4626 | **0.048** |
| AGAG-ACCE | Coral-Coral | Cor-Cor | 1.4500 | 0.4441 | 7.1906 | **0.020** |
| AGAG-ACCO | Coral-Feces | Cor-H | 1.4170 | 0.3435 | 5.2326 | **0.012** |
| AGAG-CHCA | Coral-Feces | Cor-F | 0.7189 | 0.2031 | 2.5491 | 0.070 |
| AGAG-CONA | Coral-Coral | Cor-Cor | 0.8795 | 0.2146 | 2.7321 | 0.082 |
| AGAG-CHST | Coral-Feces | Cor-F | 0.6477 | 0.2046 | 2.3150 | 0.062 |
| AGAG-DILA | Coral-Coral | Cor-Cor | 1.0238 | 0.2817 | 3.9223 | **0.044** |
| AGAG-MOCA | Coral-Coral | Cor-Cor | 0.2374 | 0.0999 | 1.2205 | 0.362 |
| AGAG-ORAN | Coral-Coral | Cor-Cor | 0.0875 | 0.0263 | 0.2704 | 0.894 |
| AGAG-POAS | Coral-Coral | Cor-Cor | 0.9584 | 0.2404 | 3.1641 | **0.044** |
| AGAG-SPAU | Coral-Feces | Cor-F | 1.0725 | 0.2360 | 3.0894 | **0.025** |
| AGAG-SPVI | Coral-Feces | Cor-F | 1.2049 | 0.2800 | 3.8880 | **0.025** |
| ACBA-ACCE | Feces-Coral | H-Cor | 1.7693 | 0.5037 | 9.1336 | **0.014** |
| ACBA-ACCO | Coral-Feces | Cor-H | 0.7778 | 0.2278 | 2.9502 | **0.029** |
| ACBA-CHCA | Feces-Feces | H-F | 0.9640 | 0.2596 | 3.5067 | **0.032** |
| ACBA-CONA | Feces-Coral | H-Cor | 1.2188 | 0.2791 | 3.8722 | **0.012** |
| ACBA-CHST | Feces-Feces | H-F | 0.4438 | 0.1535 | 1.6323 | 0.213 |

| | | | | | | |
|---|---|---|---|---|---|---|
| ACBA-DILA | Feces-Coral | H-Cor | 1.5398 | 0.3775 | 6.0651 | **0.013** |
| ACBA-MOCA | Feces-Coral | H-Cor | 1.1520 | 0.3577 | 6.1266 | **0.013** |
| ACBA-ORAN | Feces-Coral | H-Cor | 0.6374 | 0.1677 | 2.0146 | 0.083 |
| ACBA-POAS | Feces-Coral | H-Cor | 0.9054 | 0.2344 | 3.0614 | **0.025** |
| ACBA-SPAU | Feces-Feces | H-F | 0.5813 | 0.1460 | 1.7096 | 0.127 |
| ACBA-SPVI | Feces-Feces | H-F | 0.3992 | 0.1165 | 1.3184 | 0.270 |
| ACCE-ACCO | Coral-Feces | Cor-H | 1.8888 | 0.5956 | 13.2554 | **0.012** |
| ACCE-CHCA | Coral-Feces | Cor-F | 1.8711 | 0.5729 | 12.0727 | **0.013** |
| ACCE-CONA | Coral-Coral | Cor-Cor | 1.4676 | 0.4500 | 7.3650 | **0.022** |
| ACCE-CHST | Coral-Feces | Cor-F | 1.4599 | 0.5719 | 10.6881 | **0.025** |
| ACCE-DILA | Coral-Coral | Cor-Cor | 2.1711 | 0.6470 | 16.4927 | **0.012** |
| ACCE-MOCA | Coral-Coral | Cor-Cor | 2.5867 | 0.7836 | 36.2187 | **0.012** |
| ACCE-ORAN | Coral-Coral | Cor-Cor | 1.0000 | 0.3559 | 4.9723 | 0.070 |
| ACCE-POAS | Coral-Coral | Cor-Cor | 0.6395 | 0.2851 | 3.5895 | 0.070 |
| ACCE-SPAU | Coral-Feces | Cor-F | 1.7410 | 0.4597 | 7.6585 | **0.013** |
| ACCE-SPVI | Coral-Feces | Cor-F | 1.7968 | 0.5178 | 9.6628 | **0.014** |
| ACCO-CHCA | Feces-Feces | H-F | 0.7757 | 0.2532 | 3.3902 | 0.052 |
| ACCO-CONA | Feces-Coral | H-Cor | 0.9939 | 0.2700 | 3.6995 | **0.021** |
| ACCO-CHST | Feces-Feces | H-F | 1.2830 | 0.3925 | 5.8149 | **0.012** |
| ACCO-DILA | Feces-Coral | H-Cor | 1.0636 | 0.3386 | 5.1187 | **0.014** |
| ACCO-MOCA | Feces-Coral | H-Cor | 2.3089 | 0.5896 | 15.8017 | **0.012** |
| ACCO-ORAN | Feces-Coral | H-Cor | 1.0296 | 0.2758 | 3.8090 | **0.014** |
| ACCO-POAS | Feces-Coral | H-Cor | 1.0192 | 0.2899 | 4.0827 | **0.022** |
| ACCO-SPAU | Feces-Feces | H-F | 0.2468 | 0.0775 | 0.8399 | 0.581 |
| ACCO-SPVI | Feces-Feces | H-F | 0.3929 | 0.1328 | 1.5307 | 0.146 |
| CHCA-CONA | Feces-Coral | C-Cor | 0.1617 | 0.0546 | 0.5776 | 0.894 |
| CHCA-CHST | Feces-Feces | F-F | 1.1071 | 0.3454 | 4.7485 | **0.013** |
| CHCA-DILA | Feces-Coral | F-Cor | 0.0613 | 0.0272 | 0.2796 | 0.911 |
| CHCA-MOCA | Feces-Coral | F-Cor | 1.5429 | 0.4729 | 9.8688 | **0.012** |
| CHCA-ORAN | Feces-Coral | F-Cor | 0.6331 | 0.1836 | 2.2486 | 0.070 |
| CHCA-POAS | Feces-Coral | F-Cor | 1.0531 | 0.2876 | 4.0364 | **0.030** |
| CHCA-SPAU | Feces-Feces | F-F | 0.5436 | 0.1512 | 1.7813 | 0.176 |
| CHCA-SPVI | Feces-Feces | F-F | 1.0155 | 0.2749 | 3.7904 | **0.018** |
| CONA-CHST | Coral-Feces | Cor-F | 1.2273 | 0.3296 | 4.4240 | **0.012** |

| | | | | | | |
|---|---|---|---|---|---|---|
| CONA-DILA | Coral-Coral | Cor-Cor | 0.1743 | 0.0631 | 0.6734 | 0.672 |
| CONA-MOCA | Coral-Coral | Cor-Cor | 1.9272 | 0.4764 | 10.0076 | **0.012** |
| CONA-ORAN | Coral-Coral | Cor-Cor | 0.7034 | 0.1796 | 2.1886 | 0.128 |
| CONA-POAS | Coral-Coral | Cor-Cor | 0.9292 | 0.2360 | 3.0898 | **0.044** |
| CONA-SPAU | Coral-Feces | Cor-F | 0.6976 | 0.1682 | 2.0220 | 0.082 |
| CONA-SPVI | Coral-Feces | Cor-F | 1.0986 | 0.2631 | 3.5697 | **0.014** |
| CHST-DILA | Feces-Coral | F-Cor | 1.6139 | 0.4609 | 7.6930 | **0.014** |
| CHST-MOCA | Feces-Coral | F-Cor | 0.8729 | 0.3811 | 6.1579 | **0.012** |
| CHST-ORAN | Feces-Coral | F-Cor | 0.4325 | 0.1468 | 1.5485 | 0.270 |
| CHST-POAS | Feces-Coral | F-Cor | 0.9341 | 0.2882 | 3.6446 | **0.025** |
| CHST-SPAU | Feces-Feces | F-F | 0.9073 | 0.2481 | 2.9701 | **0.028** |
| CHST-SPVI | Feces-Feces | F-F | 0.6452 | 0.2135 | 2.4428 | 0.062 |
| DILA-MOCA | Coral-Coral | Cor-Cor | 2.0102 | 0.5711 | 14.6481 | **0.014** |
| DILA-ORAN | Coral-Coral | Cor-Cor | 0.9476 | 0.2667 | 3.6371 | **0.044** |
| DILA-POAS | Coral-Coral | Cor-Cor | 1.4030 | 0.3690 | 5.8489 | **0.021** |
| DILA-SPAU | Coral-Feces | Cor-F | 0.7808 | 0.2156 | 2.7478 | **0.032** |
| DILA-SPVI | Coral-Feces | Cor-F | 1.4622 | 0.3719 | 5.9223 | **0.014** |
| MOCA-ORAN | Coral-Coral | Cor-Cor | 0.4754 | 0.1821 | 2.4496 | 0.088 |
| MOCA-POAS | Coral-Coral | Cor-Cor | 1.9384 | 0.5013 | 11.0584 | **0.012** |
| MOCA-SPAU | Coral-Feces | Cor-F | 1.8716 | 0.4412 | 8.6838 | **0.014** |
| MOCA-SPVI | Coral-Feces | Cor-F | 1.8860 | 0.4855 | 10.3811 | **0.012** |
| ORAN-POAS | Coral-Coral | Cor-Cor | 0.6437 | 0.1755 | 2.1285 | 0.124 |
| ORAN-SPAU | Coral-Feces | Cor-F | 0.8563 | 0.1981 | 2.4702 | **0.014** |
| ORAN-SPVI | Coral-Feces | Cor-F | 0.9344 | 0.2319 | 3.0197 | **0.018** |
| POAS-SPAU | Coral-Feces | Cor-F | 0.9163 | 0.2194 | 2.8108 | **0.025** |
| POAS-SPVI | Coral-Feces | Cor-F | 0.8193 | 0.2210 | 2.8374 | **0.035** |
| SPAU-SPVI | Feces-Feces | F-F | 0.2869 | 0.0793 | 0.8616 | 0.535 |

B)

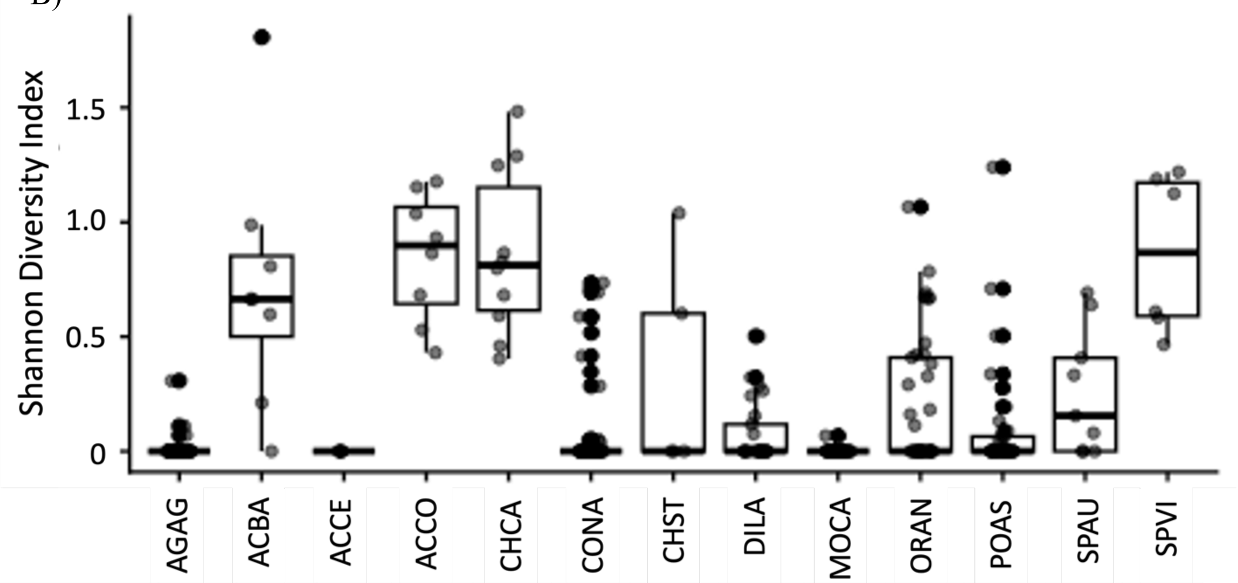


| | | | | | | | | | | | | | |
|---|---|---|---|---|---|---|---|---|---|---|---|---|---|
| **Shannon Mean** | **0.012** | **0.717** | **0.0** | **0.851** | **0.865** | **0.096** | **0.328** | **0.079** | **0.002** | **0.209** | **0.087** | **0.256** | **0.865** |

**Figure S4.** Mean relative abundances of hits to five genera of Symbiodiniaceae (based on the internal transcribed spacer-2 (ITS-2) region of rDNA) in the tissues of corals and the feces of fish from Pacific and Caribbean reefs. Species of coral and fish are listed by region, then foraging group: Caribbean coral (CCor), Caribbean herbivore/detritivore (CH), Caribbean facultative corallivore (CF), Pacific coral (PCor), Pacific herbivore/detritivore (PH), Pacific facultative corallivore (PF), Pacific obligate corallivore (PO), Caribbean facultative corallivore (CF), and Pacific facultative corallivore (PF). ACBA – *Acanthurus bahianus,* ACCO – *Acanthurus coeruleus,* ACHY – *Acropora hyacinthus,* AGAG – *Agaricia agaricites,* AMSC – *Amanses scopas,* CHCA – *Chaetodon capistratus,* CHCI – *Chaetodon citrinellus,* CHLU – *Chaetodon lunulatus,* CHOR – *Chaetodon ornatissimus,* CHPE – *Chaetodon pelewensis,* CHRE – *Chaetodon reticulatus,* CHSP – *Chlolurus spilurus,* CHST – *Chaetodon striatus,* CONA – *Colpophyllia natans,* CTFL – *Ctenochaetus flavicauda,* CTST – *Ctenochaetus striatus,* DILA – *Diploria labyrinthiformis,* MASP – *Madracis* sp., MISP – *Millepora* sp., MOCA – *Montastraea cavernosa,* MOSP – *Montipora* sp., ORAN – *Orbicella annularis,* ORFA – *Orbicella faveolata,* ORFR – *Orbicella franksi,* POAS – *Porites astreoides,* POLO – *Porites lobata,* POSP – *Pocillopora* sp., SISI – *Siderastrea siderea,* SPAU – *Sparisoma aurofrenatum,* and SPVI – *Sparisoma viride.*

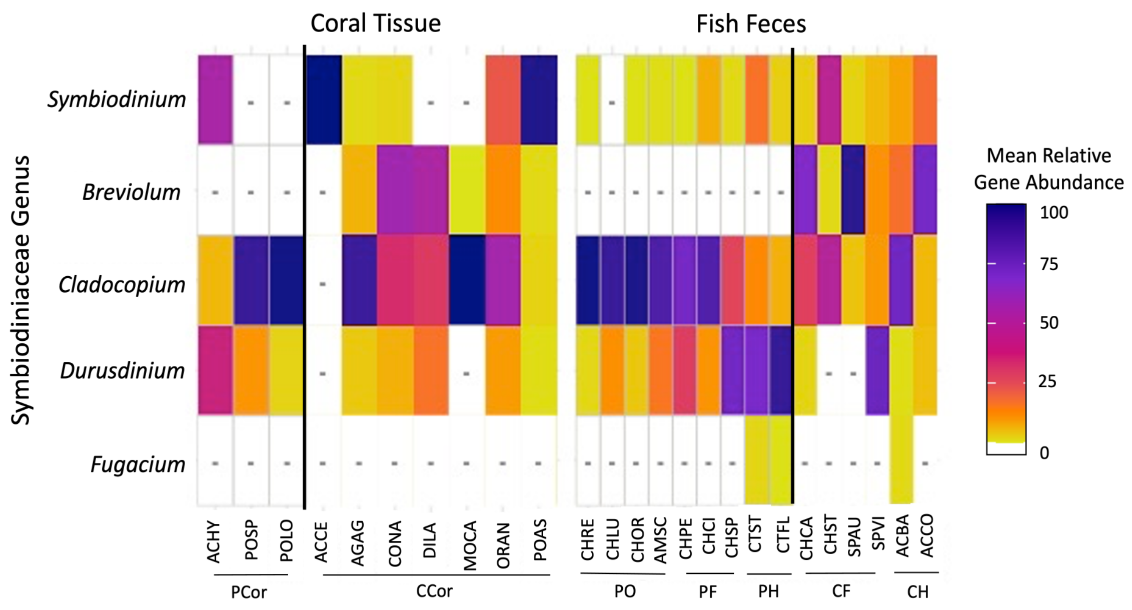

**Table S10.** Results from a pairwise PERMANOVA on the regional differences between Symbiodiniaceae internal transcribed spacer-2 (ITS-2) sequences found in the feces of reef fish and tissues of coral from Caribbean and Pacific reefs. Significant *p*-values (<0.05) are bolded. Caribbean coral – CCor, Caribbean herbivore/detritivore – CH, Caribbean facultative corallivore – CF, Pacific coral – PCor, Pacific facultative corallivore – PF, Pacific herbivore/detritivore – PH, Pacific obligate corallivore – PO. ACBA – *Acanthurus bahianus,* ACCO – *Acanthurus coeruleus,* ACHY – *Acropora hyacinthus*, AGAG – *Agaricia agaricites,* AMSC - *Amanses scopas,* CHCA – *Chaetodon capistratus,* CHCI – *Chaetodon citrinellus,* CHLU – *Chaetodon lunulatus,* CHOR – *Chaetodon ornatissimus,* CHPE – *Chaetodon pelewensis,* CHRE – *Chaetodon reticulatus,* CHSP – *Chlolurus spilurus,* CHST – *Chaetodon striatus,* CONA – *Colpophyllia natans,* CTFL – *Ctenochaetus flavicauda,* CTST – *Ctenochaetus striatus,* DILA – *Diploria labyrinthiformis,* MASP – *Madracis* sp., MISP – *Millepora* sp., MOCA – *Montastraea cavernosa,* MOSP – *Montipora* sp., ORAN – *Orbicella annularis,* ORFA – *Orbicella faveolata,* ORFR – *Orbicella franksi,* POAS – *Porites astreoides,* POLO – *Porites lobata,* POSP – *Pocillopora* sp., SISI – *Siderastrea siderea,* SPAU – *Sparisoma aurofrenatum,* and SPVI – *Sparisoma viride.* Overall PERMANOVA test results for Symbiodiniaceae ITS-2 sequences in coral tissues across regions: df = 1.294, F = 6.524, *p* = 0.001. Overall PERMANOVA test results for Symbiodinaiceae assemblages in fish feces across regions: df = 1.403, F = 26.596, *p* = 0.001.

| Species Comparison | Forage by Region | Sum of Squares | $R^2$ | F statistic | Adjusted *p*-value |
|---|---|---|---|---|---|
| ACBA – AMSC | CH-PO | 1.227 | 0.273 | 4.873 | **0.005** |
| ACBA – CHCI | CH-PF | 1.233 | 0.322 | 5.692 | **0.003** |
| ACBA – CHLU | CH-PO | 1.286 | 0.291 | 5.735 | **0.003** |
| ACBA – CHOR | CH-PO | 2.009 | 0.403 | 13.483 | **0.001** |
| ACBA – CHPE | CH-PF | 1.601 | 0.362 | 7.943 | **0.002** |
| ACBA – CHRE | CH-PO | 1.783 | 0.379 | 10.4 | **0.001** |
| ACBA – CHSP | CH-PF | 1.799 | 0.356 | 7.746 | **0.001** |
| ACBA – CTFL | CH-PH | 2.161 | 0.462 | 11.143 | **0.001** |
| ACBA – CTST | CH-PH | 1.528 | 0.359 | 6.737 | **0.001** |
| ACBA – ACHY | CH-PCor | 1.667 | 0.222 | 5.131 | **0.002** |
| ACBA – POSP | CH-PCor | 3.251 | 0.275 | 19.344 | **0.001** |
| ACBA – POLO | CH-PCor | 2.014 | 0.408 | 12.38 | **0.001** |
| ACCO – AMSC | CH-PO | 2.242 | 0.488 | 12.384 | **0.001** |
| ACCO – CHCI | CH-PF | 2.313 | 0.579 | 16.532 | **0.001** |
| ACCO – CHLU | CH-PO | 2.583 | 0.538 | 16.294 | **0.001** |
| ACCO – CHOR | CH-PO | 3.623 | 0.637 | 35.155 | **0.001** |
| ACCO – CHPE | CH-PF | 2.802 | 0.596 | 20.629 | **0.001** |
| ACCO – CHRE | CH-PO | 3.322 | 0.625 | 28.302 | **0.001** |

| | | | | | |
|---|---|---|---|---|---|
| ACCO – CHSP | CH-PF | 2.552 | 0.523 | 15.329 | **0.002** |
| ACCO – CTFL | CH-PH | 2.651 | 0.623 | 21.521 | **0.002** |
| ACCO – CTST | CH-PH | 2.013 | 0.528 | 13.402 | **0.001** |
| ACCO – ACHY | CH-PCor | 2.129 | 0.302 | 7.777 | **0.001** |
| ACCO – POSP | CH-PCor | 3.144 | 0.54 | 21.155 | **0.001** |
| ACCO – POLO | CH-PCor | 3.368 | 0.451 | 18.069 | **0.001** |
| CHCA – AMSC | CF-PO | 1.541 | 0.328 | 7.33 | **0.001** |
| CHCA – CHCI | CF-PF | 1.532 | 0.382 | 8.649 | **0.001** |
| CHCA – CHLU | CF-PO | 1.716 | 0.362 | 9.095 | **0.001** |
| CHCA – CHOR | CF-PO | 2.449 | 0.461 | 18.824 | **0.001** |
| CHCA – CHPE | CF-PF | 1.856 | 0.407 | 10.99 | **0.001** |
| CHCA – CHRE | CF-PO | 2.187 | 0.439 | 14.864 | **0.001** |
| CHCA – CHSP | CF-PF | 2.202 | 0.413 | 11.251 | **0.001** |
| CHCA – CTFL | CF-PH | 2.678 | 0.527 | 16.725 | **0.001** |
| CHCA – CTST | CF-PH | 2.086 | 0.445 | 11.221 | **0.001** |
| CHCA – ACHY | CF-PCor | 2.251 | 0.282 | 7.857 | **0.001** |
| CHCA – POSP | CF-PCor | 2.071 | 0.373 | 11.921 | **0.001** |
| CHCA – POLO | CF-PCor | 2.346 | 0.455 | 16.709 | **0.001** |
| CHST – AMSC | CF-PO | 1.075 | 0.449 | 8.966 | **0.001** |
| CHST – CHCI | CF-PF | 0.545 | 0.319 | 4.231 | **0.007** |
| CHST – CHLU | CF-PO | 0.421 | 0.198 | 2.724 | **0.048** |
| CHST – CHOR | CF-PO | 0.965 | 0.3885 | 10.644 | **0.003** |
| CHST – CHPE | CF-PF | 0.878 | 0.389 | 6.989 | **0.001** |
| CHST – CHRE | CF-PO | 0.836 | 0.362 | 7.932 | **0.008** |
| CHST – CHSP | CF-PF | 1.307 | 0.419 | 7.939 | **0.002** |
| CHST – CTFL | CF-PH | 1.823 | 0.628 | 16.866 | **0.002** |
| CHST – CTST | CF-PH | 1.153 | 0.474 | 8.096 | **0.005** |
| CHST – ACHY | CF-PCor | 1.261 | 0.222 | 4.29 | **0.006** |
| CHST – POSP | CF-PCor | 0.752 | 0.259 | 5.238 | **0.003** |
| CHST – POLO | CF-PCor | 1.102 | 0.426 | 11.113 | **0.002** |
| SPAU – AMSC | CF-PO | 2.078 | 0.365 | 8.049 | **0.001** |
| SPAU – CHCI | CF-PF | 2.225 | 0.431 | 9.838 | **0.002** |
| SPAU – CHLU | CF-PO | 2.377 | 0.406 | 10.246 | **0.001** |
| SPAU – CHOR | CF-PO | 3.427 | 0.508 | 21.668 | **0.001** |
| SPAU – CHPE | CF-PF | 2.608 | 0.452 | 12.373 | **0.001** |
| SPAU – CHRE | CF-PO | 3.052 | 0.484 | 16.874 | **0.001** |

| | | | | | |
|---|---|---|---|---|---|
| SPAU – CHSP | CF-PF | 2.372 | 0.398 | 9.905 | **0.001** |
| SPAU – CTFL | CF-PH | 2.522 | 0.468 | 12.36 | **0.001** |
| SPAU – CTST | CF-PH | 2.069 | 0.403 | 8.784 | **0.001** |
| SPAU – ACHY | CF-PCor | 2.037 | 0.248 | 6.253 | **0.001** |
| SPAU – POSP | CF-PCor | 2.919 | 0.426 | 14.092 | **0.001** |
| SPAU – POLO | CF-PCor | 3.241 | 0.498 | 18.842 | **0.001** |
| SPVI – AMSC | CF-PO | 1.423 | 0.371 | 6.483 | **0.002** |
| SPVI – CHCI | CF-PF | 1.557 | 0.472 | 8.949 | **0.004** |
| SPVI – CHLU | CF-PO | 1.627 | 0.416 | 8.565 | **0.001** |
| SPVI - CHOR | CF-PO | 2.421 | 0.533 | 20.545 | **0.001** |
| SPVI – CHPE | CF-PF | 1.804 | 0.479 | 11.035 | **0.001** |
| SPVI – CHRE | CF-PO | 2.217 | 0.519 | 16.176 | **0.001** |
| SPVI – CHSP | CF-PF | 1.479 | 0.382 | 7.425 | **0.002** |
| SPVI – CTFL | CF-PH | 1.581 | 0.488 | 10.463 | **0.002** |
| SPVI - CTST | CF-PH | 1.071 | 365 | 5.749 | **0.007** |
| SPVI – ACHY | CF-PCor | 1.339 | 0.212 | 4.295 | **0.005** |
| SPVI – POSP | CF-PCor | 2.007 | 0.423 | 11.739 | **0.001** |
| SPVI – POLO | CF-PCor | 2.418 | 0.539 | 18.705 | **0.001** |
| AGAG – ACHY | CCor-PCor | 4.793 | 0.355 | 30.211 | **0.001** |
| AGAG – POSP | CCor-PCor | 0.361 | 0.053 | 3.068 | **0.037** |
| AGAG - POLO | CCor-PCor | 0.04 | 0.007 | 0.381 | 0.916 |
| ACCE - ACHY | CCor-PCor | 0.498 | 0.101 | 1.69 | 0.151 |
| ACCE - POSP | CCor-PCor | 1.913 | 0.469 | 13.231 | **0.003** |
| ACCE - POLO | CCor-PCor | 2.051 | 0.577 | 20.486 | **0.004** |
| CONA - ACHY | CCor-PCor | 3.588 | 0.166 | 12.347 | **0.001** |
| CONA – POSP | CCor-PCor | 3.331 | 0.174 | 13.104 | **0.001** |
| CONA - POLO | CCor-PCor | 3.667 | 0.195 | 15.063 | **0.001** |
| DILA – ACHY | CCor-PCor | 2.672 | 0.199 | 8.722 | **0.001** |
| DILA – POSP | CCor-PCor | 3.051 | 0.265 | 12.612 | **0.001** |
| DILA - POLO | CCor-PCor | 3.525 | 0.311 | 15.815 | **0.001** |
| MOCA – ACHY | CCor-PCor | 5.434 | 0.509 | 50.785 | **0.001** |
| MOCA – POSP | CCor-PCor | 0.485 | 0.139 | 7.956 | 0.002 |
| MOCA - POLO | CCor-PCor | 0.033 | 0.014 | 0.698 | 0.43 |
| ORAN - ACHY | CCor-PCor | 2.089 | 0.127 | 6.866 | **0.001** |
| ORAN – POSP | CCor-PCor | 0.623 | 0.049 | 2.431 | **0.062** |
| ORAN - POLO | CCor-PCor | 0.94 | 0.076 | 3.885 | **0.008** |

| | | | | | |
|---|---|---|---|---|---|
| POAS – ACHY | CCor-PCor | 1.442 | 0.129 | 8.138 | **0.001** |
| POAS – POSP | CCor-PCor | 6.835 | 0.477 | 50.194 | **0.001** |
| POAS - POLO | CCor-PCor | 6.835 | 0.477 | 50.194 | **0.001** |
